# Associations of Socioeconomic Status With Cardiorenal Metabolic Multimorbidity: Evidence From the UK Biobank Cohort

**DOI:** 10.1016/j.jacadv.2026.102646

**Published:** 2026-03-13

**Authors:** Zhiran Guo, Yaguan Zhou, Yue Zhang, Xiaolin Xu

**Affiliations:** a School of Public Health, The Second Affiliated Hospital, Zhejiang University School of Medicine, Hangzhou, Zhejiang, China; b The Key Laboratory of Intelligent Preventive Medicine of Zhejiang Province, Hangzhou, China; c School of Public Health, Faculty of Medicine, The University of Queensland, Brisbane, Australia

**Keywords:** cardio-renal metabolic disorder, cardio-renal metabolic multimorbidity, multi-state model, socioeconomic status

## Abstract

**Background:**

Cardiorenal metabolic multimorbidity (CRMM), defined as the coexistence of ≥2 conditions among cardiovascular disease, type 2 diabetes, and chronic kidney disease, imposes a substantial global health burden. Evidence linking socioeconomic status (SES) to CRMM and its progression remains limited, despite SES associations with individual cardiorenal metabolic diseases (CRMDs).

**Objectives:**

The objective of the study was to examine the associations of SES with CRMM incidence and progression.

**Methods:**

This prospective cohort study included participants in the UK Biobank. SES (income, education, and employment) was categorized into 3 levels via latent class analysis and summed score. Cox regression models were employed to examine the association between SES and the risk of CRMM, whereas multistate modeling evaluated the dynamic progression of CRMM associated with SES.

**Results:**

During follow-up, 5,643 participants developed CRMM. Compared to high SES, low SES was independently associated with significantly elevated risks of CRMM (HR: 2.03; 95% CI: 1.82 to 2.27). Socioeconomic disparities were observed across all disease transitions, with low SES increasing progression risks from free of CRMD to first CRMD (HR: 1.45; 95% CI: 1.10-1.50), to CRMM (HR: 1.51; 95% CI: 1.43-1.49), and from existing cardiovascular disease (HR: 1.22; 95% CI: 1.12-1.33) or type 2 diabetes (HR: 1.48; 95% CI: 1.19-1.85) to CRMM. Consistent associations were confirmed using the summed SES score.

**Conclusions:**

Our study establishes SES as a pivotal determinant of CRMM incidence and progression. This underscores the necessity of integrating SES into public health strategies to mitigate the CRMM burden, even in individuals with 1 CRMD.

Cardiorenal metabolic diseases (CRMDs), including cardiovascular disease (CVD), type 2 diabetes (T2D), and chronic kidney disease (CKD) contribute significantly to mortality and disability, representing a leading cause of disease burden worldwide.^1^, ^2^, ^3^ Crucially, pathophysiological mechanisms and risk factors, including hyperglycemia, dyslipidemia, hypertension, and obesity, not only drive the development of each individual specific disease, but also exhibit system interdependence.^4^, ^5^, ^6^ Dysfunction in 1 system may amplify impairment in others, accelerating disease progression and worsening clinical outcomes.^7^, ^8^, ^9^ This interplay underlies cardiorenal metabolic multimorbidity (CRMM), described by existence of two or more conditions among CVD, T2D, and CKD in 1 person.^10^, ^11^, ^12^ Epidemiological data highlight the substantial public health burden of CRMM, affecting over 1/4 of U.S. adults during 2015 to 2020, and currently accounts for approximately 1 in every 3 deaths.^12^, ^13^, ^14^, ^15^, ^16^

Socioeconomic status (SES), encompassing factors such as income, education, and occupation, is a crucial social determinant of many health outcomes, which directly determines the quality and accessibility of health care.^17^ Accumulative evidence has demonstrated that socioeconomic inequalities existed in CVD, T2D, and CKD individually, with lower SES consistently associated with higher incidence and worse prognosis of these diseases.^18^, ^19^, ^20^, ^21^, ^22^ For instance, a national study of 48,190 participants demonstrated that individuals in the lowest SES group had a 2.32-fold higher risk of all-cause mortality and a 1.59-fold higher incidence of CVD compared to those in the highest SES group.^23^ Among individuals with diabetes, those experiencing socioeconomic deprivation had a significantly higher mortality (HR: 1.32; 95% CI: 1.26-1.37). Simultaneously, epidemiological data from multiple regions have confirmed that socioeconomic disadvantage is independently linked to increasing burden of multimorbidity.^24^, ^25^, ^26^, ^27^ A projection study indicated that by 2049, the prevalence of multimorbidity will rise by approximately one-third (34%) compared to 2019. This increase is disproportionately concentrated among the most socioeconomically disadvantaged populations, with cumulative incident cases expected to exceed 1.07 million, signifying an urgent public health crisis.^28^

Despite the well-established link between low SES and increased risk of both CRMDs and multimorbidity, evidence specifically estimating the association between SES and CRMM remains lacking. Given the distinct pathophysiological and clinical characteristics of CRMM,^6^ SES likely exerts amplified, rather than merely additive, effects on disease progression. An exploration of the association between SES and CRMM is crucial for identifying high-risk populations and formulating more precise interventions.

This study aimed to address the above evidence gap. Leveraging data from 394,208 participants with a median follow-up of approximately 12.6 years (Q1-Q3: 11.9-13.3) in the UK Biobank, we investigated the relationship between SES and CRMM, as well as its progression over time.

## Methods

### Study design and participants

The study used data from the UK Biobank, a large-scale prospective cohort study launched in the UK between 2006 and 2010 that recruited over 500,000 participants aged 40 to 69 years. All participants provided written informed consent, and ethical approval for the study was granted by the North West Multicentre Research Ethics Committee. Sociodemographic characteristics, lifestyle factors, and health-related parameters were assessed at baseline through self-administered touchscreen questionnaires and standardized physical measurements. National health records (hospital inpatient data, coded primary care data, cancer and death registry data) are accessible for all participants through National Health Service Digital linkages.^29^

Among 502,490 participants initially recruited, we sequentially excluded: 1) those with missing socioeconomic factors (n = 79,855); 2) prevalent cases of CVD, T2D, or CKD at cohort entry (n = 27,387); and 3) individuals lost to follow-up (n = 1,040). The final analytical cohort comprised 394,208 participants (see Supplemental Figure 1).

### Assessment of SES

Individual-level SES was assessed through 3 primary indicators: the total household income level before tax, education qualification, and employment status.^30^^,^^31^ The total household income level before tax was categorized as: 1) <£18,000; 2) £18,000–£30,999; 3) £31,000–£51,999; 4) £52,000–£100,000; and 5) >£100,000. Participants indicated their level of education by selecting one of the following options: 1) college or university degree; A-Level, Advanced Subsidiary Level (AS-Level), or equivalent; 3) O-Level, General Certificate of Secondary Education (GCSE), or equivalent; 4) Certificate of Secondary Education (CSE) or equivalent; 5) National Vocational Qualification (NVQ), Higher National Diploma (HND), Higher National Certificate (HNC), or equivalent; 6) other professional qualifications; and 7) none of the above. Employment status was dichotomized as employed (in paid employment or self-employed, retired, doing unpaid or voluntary, or full or part-time student) and unemployed (looking after home and/or family, unable to work because of sickness or disability, unemployed, none of the above, or prefer not to answer). Variable specifications are provided in Supplemental Text 1.

We employed 2 distinct methods to assess individual SES. Initially, we used latent class analysis (LCA) to derive a composite variable from the 3 indicators (total household income level before tax, education qualification, and employment status) for assessing SES.^30^ The LCA was conducted using the R package poLCA, which is a statistical method that uses multiple observed categorical variables to derive a latent variable comprising mutually exclusive classes, thereby clustering individuals into distinct types based on their response patterns.^32^ The number of latent classes were selected according to the parameters of the model and the practical implications, as detailed in Supplemental Text 1 (Supplemental Material), which also includes a distribution plot of the different SES classes. Based on the item response probabilities, we identified 3 distinct latent classes, corresponding to high, medium, and low SES levels. Subsequently, we scored each of the 3 aforementioned indicators individually and summed the scores to evaluate SES,^33^ which was then categorized into high (3), medium (1-2), and low (0) levels. Detailed information on these methods is provided in the Supplemental Material.

In addition to individual-level SES, we employed the Townsend Deprivation Index (TDI) to evaluate area-level SES, which incorporates 4 domain indicators: car ownership, household overcrowding, owner-occupied housing, and unemployment rate. Higher TDI scores denote lower area-level SES. We categorized the TDI into 3 tertile-based deprivation groups: T_1_ (low socioeconomic deprivation), T_2_ (medium socioeconomic deprivation), and T_3_ (high socioeconomic deprivation).^34^

### Follow-up and outcome assessment

The primary outcome was CRMM, defined as the development of at least two of the following diseases: CVD, T2D, and CKD. Specifically, CVD includes coronary heart disease, atrial fibrillation, heart failure, peripheral artery disease, and stroke. The date of the occurrence of CVD, T2D, CKD, or death after baseline was determined by linking hospital episode statistics from England, Scotland, and Wales with records from national death registries. CRMDs were defined using the International Classification of Diseases, 10th Revision codes (Supplemental Text 3). Person-time (in years) was calculated from baseline until the occurrence of the study outcome or the end of follow-up, whichever occurred first.

### Assessment of covariates

Covariates were collected at baseline, including sociodemographic characteristics (age, sex, and ethnicity), anthropometric information (body mass index), lifestyle factors (current smoking and drinking status, physical activity, dietary information and sleep duration), and medical history (chronic obstructive pulmonary disease [COPD] and cancer at baseline). Participants with missing information on these covariates were assigned to a separate “unknown” category. Body mass index was categorized into underweight (<18.5 kg/m^2^), normal weight (18.5-24.9 kg/m^2^), overweight (25.0-29.9 kg/m^2^), and obese (≥30.0 kg/m^2^) groups.^35^ Physical activity was assessed using weekly metabolic equivalent task minutes and further stratified into tertiles (low, medium, and high). Diet information was evaluated using updated cardiovascular health dietary recommendations that considered adequate intake of fruits, vegetables, whole grains, and fish, along with reduced consumption of refined grains, processed meats, and unprocessed red meats. A healthy diet was defined as meeting at least 4 of these recommendations.^36^ Sleep duration was classified as <7 h/day, 7 to 9 h/day, and >9 h/day^37^(Supplemental Text 2). Hypertension was defined by blood pressure measurement (systolic blood pressure ≥140 mm Hg and/or diastolic blood pressure ≥90 mm Hg). Dyslipidemia was defined by total cholesterol (≥5 mmol/L) or low-density lipoprotein cholesterol (≥3 mmol/L).^38^ Medication histories were assessed based on self-reported data.

### Statistical analyses

Baseline characteristics were summarized as the mean (SD) or median (25th-75th percentiles [Q1-Q3]) for continuous variables and the number (percentage) for categorical variables. Categorical and continuous variables were analyzed using the chi-square test and analysis of variance, respectively.

We applied Cox proportional hazards models to estimate HRs with 95% CIs for associations between SES and risks of each CRMD and CRMM as well as different CRMM patterns. Schoenfeld residual test was used to verify the assumption of proportional hazards in the Cox analysis. Survival curves were plotted using Kaplan-Meier analysis. Sequential covariate adjustment across 4 hierarchical models were performed: model 1 was the crude model; model 2 was adjusted for sociodemographic characteristics; model 3 was additionally adjusted for anthropometric measurements and lifestyle factors; and model 4 was further adjusted for hypertension, dyslipidemia, medical history, COPD, and cancer at baseline. Recognizing that death from other causes acts as a competing event that may preclude the occurrence of the cardiorenal metabolic outcomes of interest, we complemented the primary Cox models (which estimate cause-specific hazards) with analyses using the Fine-Gray subdistribution hazards model.^39^ These analyses were performed for the outcomes of incident CVD, T2D, CKD, and CRMM.

A series of exploratory and sensitivity analyses were conducted to test the robustness of our results. Firstly, we examined the relationships between income, educational attainment, employment status, and each CRMD and CRMM. Secondly, we repeated the primary analyses by; 1) excluding participants with missing covariate data; 2) imputing missing data of covariates using multiple imputation; 3) excluding outcome events that occurred in the first 180 days of follow-up; 4) repeating the competing risk analyses for single disease and CRMM; and 5) utilizing TDI as the primary measure of SES instead of the individual-level composite SES. Third, to assess the difference between SES and TDI, we quantified their correlation using Cramer's V. Fourth, we added TDI as a covariate to the fully adjusted multivariable Cox model (model 4). Finally, we conducted stratified analyses by TDI to assess whether the associations of individual SES with CVD, T2D, CKD, and CRMM were consistent across different contexts of area deprivation. We employed a multistate model^40^ to evaluate the role of SES in the dynamic transitions from free of CRMD to first CRMD, CRMM, and death. Five transitions were constructed: 1) free of CRMD to first CRMD; 2) free of CRMD to non-CRMD-related mortality; 3) first CRMD to CRMM; 4) first CRMD to all-cause mortality; and 5) CRMM to all-cause mortality. For participants with concurrent diagnoses of two or more conditions, simultaneously entering both states of first CRMD and CRMM, we implemented an adjustment to ensure temporal precedence in state transitions. Specifically, the entry time for the state of first CRMD was calculated as the diagnosis date minus 0.5 days.^41^ In sensitivity analyses examining the robustness of state transition timing, we systematically varied the interval for defining entry into the first CRMD state. Alternative intervals of 30, 180, and 365 days were examined to assess potential misclassification effects arising from diagnostic date uncertainty.

We further specified first CRMD into 3 individual diseases (CVD, T2D, and CKD) and repeated the multistate models. A total of 11 transitions were identified, which comprehensively captures the impact of socioeconomic determinants on CRMDs and their progression.

For individuals transitioning to different states on the same date, we recalculated the entry date of the preceding state using distinct time intervals (30 days, 180 days, and 365 days).

Statistical analyses were performed using R software (version 4.4.1, R Foundation for Statistical Computing) and SAS software (version 9.4; SAS Institute Inc). Two-tailed *P* < 0.05 was considered statistically significant.

## Results

### Population characteristics

Table 1 shows the baseline characteristics of the study population. The mean age of the included 394,208 participants was 55.8 (SD 8.1) years, and 181,117 (45.9%) participants were males. Based on the result of LCA, participants were stratified into three groups: high SES (n = 79,856, 20.3%), medium SES (n = 209,033, 53.0%), and low SES (n = 105,319, 26.7%). During a median of 12.6 years (Q1-Q3: 11.9-13.3) of follow-up, we documented 29,238 incident CVD cases, 14,522 incident T2D cases and 4,908 incident CKD cases. Crucially, 5,643 participants developed CRMM. All baseline characteristics differed significantly across the three LCA-defined SES strata. Compared to the high SES group, participants with low SES group were older on average and more likely to be female. Individuals with low SES had a higher prevalence of obesity, smoking, drinking and physical inactivity, and prolonged sleep duration (>9 hours). The prevalence of each chronic condition (CVD, T2D, CKD, and COPD) was significantly higher among individuals with low SES. Among participants who developed CRMM (n = 5,643), those in the low SES group comprised the largest proportion, accounting for 49.2% of those with 2 CRMDs and 56.6% of those with three CRMDs. Consistently, the baseline characteristics table using the summed-score SES classification (Supplemental Table 2) revealed a significantly higher proportion of individuals with CRMM in the low (0) SES group compared to the high (3) SES group.

**Table 1:** Baseline Characteristics of Study Participants According to Socioeconomic Status Defined by Latent Class Analysis

	Total (N = 394,208)	High SES (n = 79,856)	Medium SES (n = 209,033)	Low SES (n = 105,319)
Age (year, mean, [SD])	55.8 (8.1)	52.4 (7.2)	55.5 (8.0)	59.1 (7.7)
Sex (n, %)				
Female	213,091 (54.1)	39,586 (49.6)	111,538 (53.4)	61,967 (58.8)
Male	181,117 (45.9)	40,270 (50.4)	97,495 (46.6)	43,352 (41.2)
Ethnicity (n, %)				
White	374,872 (95.1)	76,532 (95.8)	199,782 (95.6)	98,558 (93.6)
Non-White	18,340 (4.7)	3,174 (4.0)	8,749 (4.2)	6,417 (6.1)
Unknown	996 (0.3)	150 (0.2)	502 (0.2)	344 (0.3)
Total household income level before tax (n, %)				
>£100,000	22,106 (5.6)	21,678 (27.2)	0 (0)	428 (0.4)
£52,000–£100,000	82,951 (21.0)	56,909 (71.3)	26,042 (12.5)	0 (0)
£31,000–£51,999	104,901 (26.6)	1,269 (1.6)	103,187 (49.4)	445 (0.4)
£18,000–£30,999	99,585 (25.3)	0 (0)	79,804 (38.2)	19,781 (18.8)
<£18,000	84,665 (21.5)	0 (0)	0 (0)	84,665 (80.4)
Education qualification (n, %)				
College or university degree	141,310 (35.8)	62,684 (78.5)	63,970 (30.6)	14,656 (13.9)
A-Level/AS-Level levels or equivalent	46,612 (11.8)	13,700 (17.2)	25,209 (12.1)	7,703 (7.3)
O-Level/GCSE or equivalent	84,384 (21.4)	1,995 (2.5)	62,436 (29.9)	19,953 (19.0)
CSE or equivalent	21,682 (5.5)	246 (0.3)	15,632 (7.5)	5,804 (5.5)
NVQ or HND or HNC or equivalent	25,461 (6.5)	475 (0.6)	17,808 (8.5)	7,178 (6.8)
Other professional qualifications	19,733 (5.0)	756 (1.0)	14,493 (6.9)	4,484 (4.3)
None of the above	55,026 (14.0)	0 (0)	9,485 (4.5)	45,541 (43.2)
Employment status (n, %)				
Employed	366,425 (93.0)	75,028 (94.0)	20,5731 (98.4)	85,666 (81.3)
Unemployed	27,783 (7.0)	4,828 (6.0)	3,302 (1.6)	19,653 (18.7)
TDI (n, %)				
Low	132,907 (33.7)	33,190 (41.6)	76,221 (36.5)	23,496 (22.3)
Medium	131,880 (33.5)	26,310 (32.9)	73,391 (35.1)	32,179 (30.6)
High	129,421 (32.8)	20,356 (25.5)	59,421 (28.4)	49,644 (47.1)
BMI (n, %)				
<18.5 kg/m^2^	3,737 (0.9)	647 (0.8)	1,653 (0.8)	1,437 (1.4)
18.5-24.9 kg/m^2^	128,562 (32.6)	31,469 (39.4)	67,419 (32.3)	29,674 (28.2)
25-29.9 kg/m^2^	165,762 (42.1)	32,977 (41.3)	89,343 (42.7)	43,442 (41.2)
≥30 kg/m^2^	89,918 (22.8)	13,519 (16.9)	47,170 (22.6)	29,229 (27.8)
Unknown	6,229 (1.6)	1,244 (1.6)	3,448 (1.6)	1,537 (1.5)
Healthy diet (n, %)				
Yes	178,828 (45.4)	40,321 (50.5)	94,781 (45.3)	43,726 (41.5)
No	215,380 (54.6)	39,535 (49.5)	114,252 (54.7)	61,593 (58.5)
Smoking status (n, %)				
Never	217,990 (55.3)	49,828 (62.4)	117,343 (56.1)	50,819 (48.3)
Previous	134,036 (34.0)	24,784 (31.0)	71,476 (34.2)	37,776 (35.9)
Current	41,118 (10.4)	5,153 (6.5)	19,801 (9.5)	16,234 (15.4)
Unknown	994 (0.3)	91 (0.1)	413 (0.2)	490 (0.5)
Drinking status (n, %)				
Never	14,580 (3.7)	1,451 (1.8)	6,248 (3.0)	6,881 (6.5)
Previous	12,654 (3.2)	1,470 (1.8)	5,326 (2.5)	5,858 (5.6)
Current	366,689 (93.0)	76,914 (96.3)	197,373 (94.4)	92,402 (87.7)
Unknown	285 (0.1)	21 (0)^a^	86 (0)^a^	178 (0.2)
Physical activity (n, %)				
Low	61,319 (15.6)	14,366 (18.0)	32,092 (15.4)	14,861 (14.1)
Medium	135,694 (34.4)	31,513 (39.5)	72,212 (34.5)	31,969 (30.4)
High	133,721 (33.9)	26,103 (32.7)	72,405 (34.6)	35,213 (33.4)
Unknown	63,474 (16.1)	7,874 (9.9)	32,324 (15.5)	23,276 (22.1)
Sleep duration (n, %)				
<7 h	94,485 (24.0)	16,896 (21.2)	49,063 (23.5)	28,526 (27.1)
7-9 h	292,298 (74.1)	62,510 (78.3)	157,402 (75.3)	72,386 (68.7)
>9 h	6,013 (1.5)	407 (0.5)	2,125 (1.0)	3,481 (3.3)
Unknown	1,412 (0.4)	43 (0.1)	443 (0.2)	926 (0.9)
Hypertension (n, %)				
Yes	182,469 (46.3)	30,712 (38.5)	96,919 (46.4)	54,838 (52.1)
No	211,739 (53.7)	49,144 (61.5)	112,114 (53.6)	50,481 (47.9)
Use of antihypertensive medication (n, %)				
Yes	68,155 (17.3)	8,457 (10.6)	34,250 (16.4)	25,448 (24.2)
No	326,053 (82.7)	71,399 (89.4)	174,783 (83.6)	79,871 (75.8)
Dyslipidemia (n, %)				
Yes	295,020 (74.8)	59,814 (74.9)	157,705 (75.4)	77,501 (73.6)
No	99,188 (25.2)	20,042 (25.1)	51,328 (24.6)	27,818 (26.4)
Use of lipid-lowering medications (n, %)				
Yes	52,918 (13.4)	6,758 (8.5)	25,526 (12.2)	20,634 (19.6)
No	341,290 (86.6)	73,098 (91.5)	183,507 (87.8)	84,685 (80.4)
COPD at baseline (n, %)				
Yes	7,663 (1.9)	316 (0.4)	2,651 (1.3)	4,696 (4.5)
No	386,545 (98.1)	79,540 (99.6)	206,382 (98.7)	100,623 (95.5)
Cancer at baseline (n, %)				
Yes	51,469 (13.1)	7,109 (8.9)	25,129 (12.0)	16,430 (15.6)
No	342,739 (86.9)	72,747 (91.1)	183,904 (88)	88,889 (84.4)

AS = advanced subsidiary; BMI = body mass index; COPD = chronic obstructive pulmonary disease; CSE = Certificate of Secondary Education; GCSE = General Certificate of Secondary Education; HNC = Higher National Certificate; HND = Higher National Diploma; NVQ = National Vocational Qualification; SES = socioeconomic status; TDI = Townsend Deprivation Index.

*P* values were calculated using analysis of variance and chi-square test test for continuous and categorical variables, respectively. All *P* values were <0.001.

a The value is <0.1% but rounds to 0.0% when formatted to 1 decimal place.

### Cox regression analyses

As shown in Table 2, SES demonstrated independent associations with CVD, T2D, CKD, and CRMM. Participants with low SES, as defined by LCA, exhibited significantly increased risks of developing CVD (HR: 1.31; 95% CI: 1.26-1.37), T2D (HR: 1.73; 95% CI: 1.62-1.83), and CKD (HR: 1.82; 95% CI: 1.62-2.05) compared to the high SES group. The association between SES and each CRMD followed dose-dependent patterns, with HR (95% CI) per decreasing SES level for CVD, T2D, and CKD were 1.15 (1.13, 1.17), 1.30 (1.27, 1.34), and 1.29 (1.23, 1.36). Compared to the high SES group, lower levels of SES were significantly associated with an elevated risk of CRMM, with HRs of 1.55 (95% CI: 1.40-1.73) for medium SES and 2.03 (95% CI: 1.82-2.27) for low SES, respectively, after adjusting for sociodemographic characteristics, anthropometric information, lifestyle factors, and medical history. Each decrease in SES category was associated with a 40% higher risk of CRMM (HR: 1.37; 95% CI: 1.31-1.43), as presented in Table 2 and the Central Illustration.

**Table 2:** Associations of Different Patterns of Socioeconomic Status With Cardiorenal Metabolic Diseases (N = 394,208)

	CVD (n = 29,238)		T2D (n = 14,522)		CKD (n = 4,908)		CRMM (n = 5,643)	
	Cases	HR (95% CI)	Cases	HR (95% CI)	Cases	HR (95% CI)	Cases	HR (95% CI)
LCA-defined SES								
High	3,550	1 .00 (Ref)	1,403	1.00 (Ref)	364	1.00 (Ref)	394	1.00 (Ref)
Medium	14,191	1.14 (1.10, 1.19)	6,650	1.35 (1.28, 1.43)	2,228	1.49 (1.33, 1.66)	2,436	1.55 (1.40, 1.73)
Low	11,497	1.31 (1.26, 1.37)	6,469	1.73 (1.62, 1.83)	2,316	1.82 (1.62, 2.05)	2,813	2.03 (1.82, 2.27)
Per score point	-	1.15 (1.13, 1.17)	-	1.30 (1.27, 1.34)	-	1.29 (1.23, 1.36)	-	1.37 (1.31, 1.43)
Summed SES Score								
High [3]	2,653	1.00 (Ref)	994	1.00 (Ref)	282	1.00 (Ref)	281	1.00 (Ref)
Medium [1-2]	24,703	1.17 (1.12, 1.22)	12,117	1.46 (1.37, 1.56)	4,291	1.47 (1.30, 1.66)	4,843	1.71 (1.51, 1.93)
Low [0]	1,882	1.62 (1.52, 1.72)	1,411	2.18 (2.00, 2.37)	335	2.11 (1.79, 2.48)	519	2.60 (2.24, 3.02)
Per score point	-	1.13 (1.11, 1.15)	-	1.26 (1.23, 1.30)	-	1.27 (1.22, 1.33)	-	1.32 (1.27, 1.38)

Adjusted for age at baseline, sex, ethnicity, BMI at baseline, current smoking and drinking status, healthy diet, sleep duration, physical activity, hypertension, dyslipidemia, medical history, COPD, and cancer at baseline.

All *P* values are statistically significant (*P* < 0.001).

CKD = chronic kidney disease; CRMM = cardiorenal metabolic multimorbidity; CVD = cardiovascular diseases; LCA = latent class analysis; T2D = type 2 diabetes; other abbreviation as in Table 1.

**Central Illustration:**
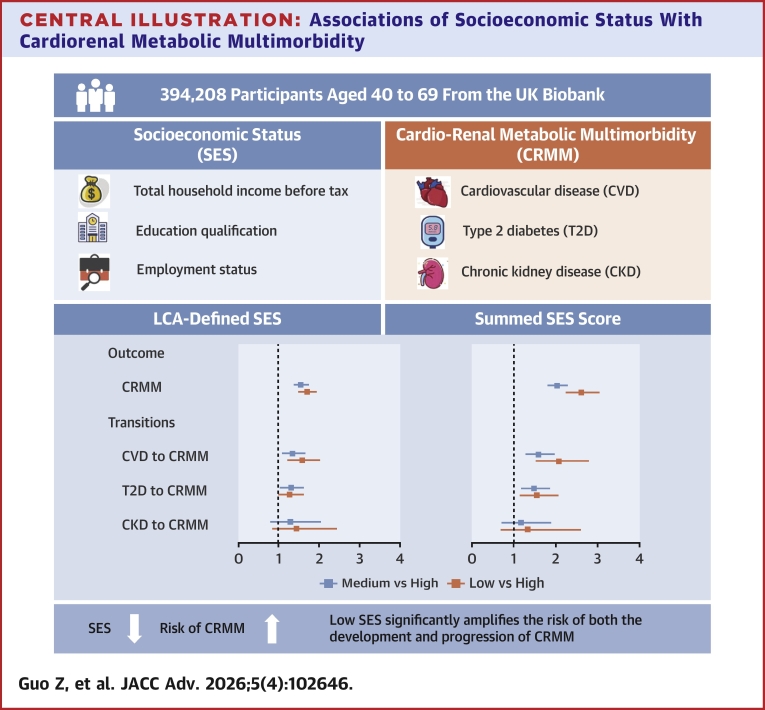
**Associations of Socioeconomic Status With Cardiorenal Metabolic Multimorbidity** This central illustration depicts key elements from a prospective UK Biobank study investigating the association between socioeconomic status (SES) and cardio-renal metabolic multimorbidity (CRMM). The study included 394,208 participants, aged 40 to 69 and free of cardiovascular disease (CVD), type 2 diabetes (T2D), and chronic kidney disease (CKD) at baseline. SES was assessed using both latent class analysis (LCA) and a summed score based on income, education, and employment. The primary outcome was incident CRMM, defined as the development of at least 2 conditions from CVD, T2D, and CKD. SES was associated with the incidence of CRMM and the progression from CVD or T2D to CRMM.

Analyses using the summed SES score yielded consistent results: individuals with low SES (0) had significantly elevated risks of CVD (HR: 1.62; 95% CI: 1.52-1.72), T2D (HR: 2.18; 95% CI: 2.00-2.37), CKD (HR: 2.11; 95% CI: 1.79-2.48), and CRMM (HR: 2.60; 95% CI: 2.24-3.02) compared with their high SES (3) counterparts. Furthermore, each one-point reduction in the summed SES score was associated with stepwise increases in risks of CVD (HR: 1.13; 95% CI: 1.11-1.15), T2D (HR: 1.26; 95% CI: 1.23-1.30), CKD (HR: 1.27; 95% CI: 1.22-1.33), and CRMM (HR: 1.32; 95% CI: 1.27-1.38). In Table 3, the strongest SES-CRMM association emerged for the triad diseases (CVD+T2D+CKD). When assessed via LCA, low SES conferred a 2.78-fold elevated risk (95% CI: 1.74-4.43) compared to high SES groups. This gradient steepened under summed SES scoring: individuals at the extreme deprivation level (score = 0) faced an approximately 3-fold risk (HR: 3.22; 95% CI: 1.77-5.84). These analyses were performed using models 1 to 4, as detailed in Supplemental Tables 3 and 4. SES components, including total household income level, education qualifications, and employment status, also presented significant associations with CVD, T2D, CKD, and CRMM. The results showed that lower income had a clear dose-response relationship with higher risks of CRMM (vs >£100,000; HR: 1.87; 95% CI: 1.52-2.29 for CRMM), unemployed individuals had higher risks than employed ones for CRMM (HR: 1.37; 95% CI: 1.25-1.50 for CRMM), and education showed weaker effects—those with no qualifications had a higher CRMM risk compared with individuals with a college/university degree (HR: 1.22; 95% CI: 1.12-1.33) (Supplemental Table 5). The Schoenfeld residual test showed no violation of the proportional hazards assumption. The Kaplan-Meier plots depicting the unadjusted associations are provided in Supplemental Figures 2 and 3 for visual reference.

**Table 3:** Associations of Different Patterns of Socioeconomic Status With Patterns of Cardiorenal Metabolic Multimorbidity (N = 394,208)

	CVD and T2D (n = 3,313)		CVD and CKD (n = 1,343)		T2D and CKD (n = 503)		CVD and T2D and CKD (n = 484)	
	Cases	HR (95% CI)	Cases	HR (95% CI)	Cases	HR (95% CI)	Cases	HR (95% CI)
LCA-defined SES								
High	255	1.00 (Ref)	88	1.00 (Ref)	31	1.00 (Ref)	20	1.00 (Ref)
Medium	1,439	1.51 (1.32, 1.73)	594	1.50 (1.19, 1.88)	213	1.54 (1.05, 2.25)	190	2.07 (1.30, 3.29)
Low	1,619	2.03 (1.77, 2.33)	661	1.82 (1.44, 2.29)	259	1.92 (1.30, 2.83)	274	2.78 (1.74, 4.43)
Per score point	-	1.39 (1.31, 1.47)	-	1.28 (1.17, 1.41)	-	1.31 (1.13, 1.53)	-	1.47 (1.25, 1.73)
Summed SES Score								
High [3]	181	1.00 (Ref)	62	1.00 (Ref)	23	1.00 (Ref)	15	1.00 (Ref)
Medium [1-2]	2,774	1.66 (1.43, 1.94)	1,196	1.67 (1.28, 2.16)	449	1.70 (1.11, 2.60)	424	2.17 (1.29, 3.66)
Low [0]	358	2.73 (2.27, 3.28)	85	2.40 (1.72, 3.36)	31	1.84 (1.06, 3.20)	45	3.22 (1.77, 5.84)
Per score point	-	1.34 (1.27, 1.40)	-	1.32 (1.21, 1.44)	-	1.18 (1.03, 1.35)	-	1.36 (1.17, 1.57)

Adjusted for age at baseline, sex, ethnicity, BMI at baseline, current smoking and drinking status, healthy diet, sleep duration, physical activity, hypertension, dyslipidemia, medical history, COPD, and cancer at baseline.

All *P* values are statistically significant (*P* < 0.05).

Abbreviation as in Tables 1 and 2.

### Multistate analyses

**Figure 1:**
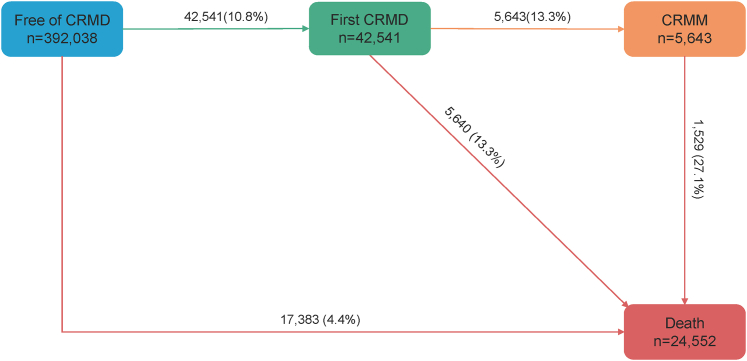
**Participants in Transition Pattern A** Pattern A refers to the transitions from baseline to first cardiorenal metabolic disease (CRMD), to cardiorenal metabolic multimorbidity (CRMM), and to death. (CRMM is defined as the coexistence of 2 or 3 CRMDs).

**Figure 2:**
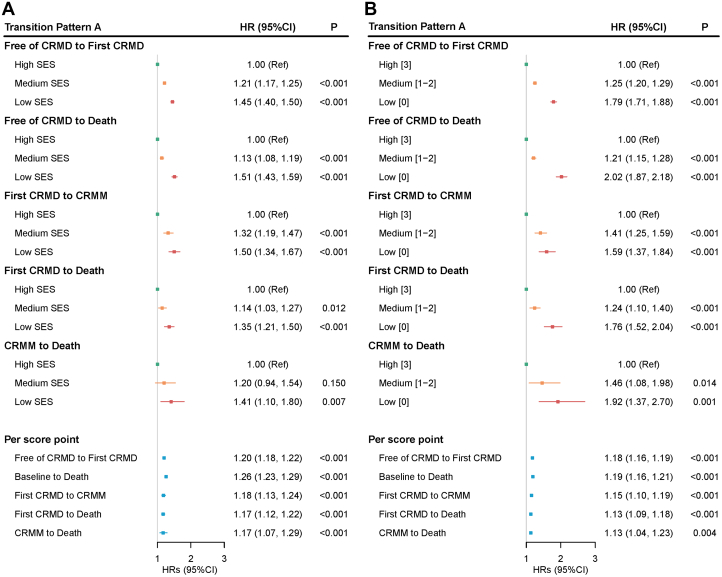
**Transition Pattern A by Socioeconomic Status** This transition analysis was conducted in a population of 394,208. (A) SES was created using latent class analysis based on income, education, and employment status. (B) SES was the sum of the points of income, education, and employment status. CRMD = cardiorenal metabolic disease, CRMM = cardiorenal metabolic multimorbidity (the coexistence of two or three CRMDs); SES = socioeconomic status.

During the follow-up period, 42,541 participants developed at least 1 CRMD, and 5,643 of these individuals further progressed to CRMM. A total of 24,552 deaths were identified during the follow-up, including 5,640 deaths transitioned from the first CRMD and 1,529 from CRMM (Figure 1). As shown in Figure 2, LCA-defined SES presented significant association with all transitions between free of CRMD and CRMM or death. Compared to high SES group, the low SES group showed increased risk of transitioning from free of CRMD to first CRMD (HR: 1.45; 95% CI: 1.40-1.50) and from first CRMD to CRMM (HR: 1.35; 95% CI: 1.21-1.50). These associations still existed when measuring the summed SES score (Figure 2).

**Figure 3:**
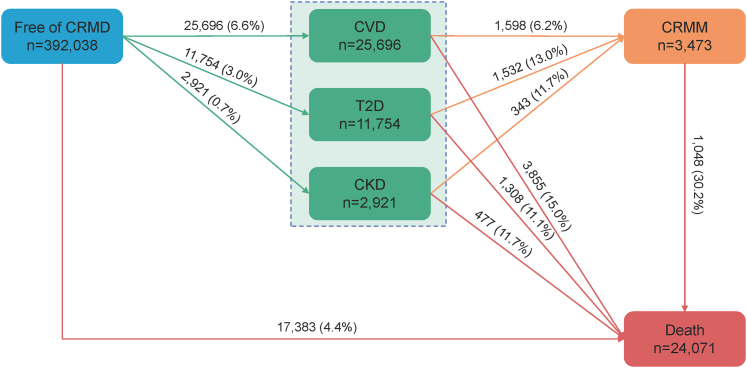
**Participants in Transition Pattern B** Pattern B refers to the transitions from baseline to one of CVD, T2D, and CKD, then to CRMM, and subsequently to death. CVD, cardiovascular diseases; T2D, type 2 diabetes; CKD, chronic kidney disease; other abbreviations as in Figure 2.

**Figure 4:**
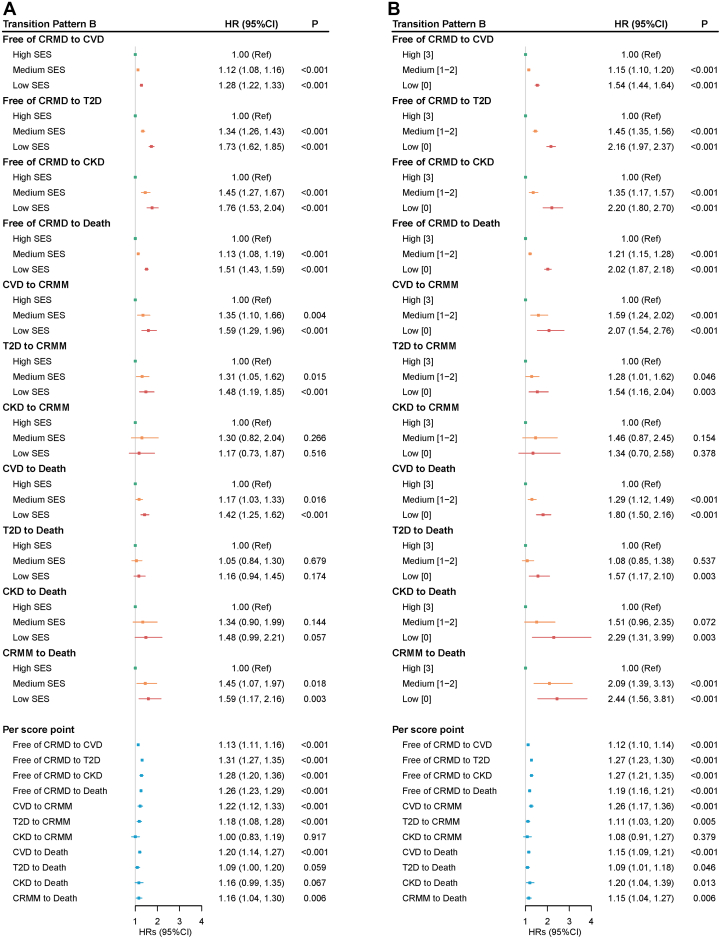
**Transition Pattern B by Socioeconomic Status** This transition analysis was conducted in a population of 392,038. (A) SES was created using latent class analysis based on income, education, and employment status. (B) SES was the sum of the points of income, education, and employment status. Abbreviations as in Figures 2 and 3.

Regarding the disease-specific transitions (2,170 participants were excluded), there were 25,696 first incident cases of CVD, 11,754 first incident cases of T2D, and 2,921 first incident cases of CKD (see Figure 3). SES remained significantly associated with most transitions between free of CRMD and CRMM or death, but to different extents. The risk estimates were highest in the associations between low SES and transitions from free of CRMD to T2D (vs high SES; HR: 1.73; 95% CI: 1.62-1.85) and to CKD (vs high SES; HR: 1.76; 95% CI: 1.53-2.04). For participants with CRMDs of CVD, T2D, and CKD, the associations between SES and transitioning to CRMM were only significant in those with CVD (low vs high SES; HR: 1.59; 95% CI: 1.29-1.96) and T2D (low vs high SES; HR: 1.48; 95% CI: 1.19-1.85). When measuring the SES score, the associations were slightly enhanced (Figure 4 and Central Illustration).

### Additional analyses

Results remained consistent with those from main analyses by excluding observations with missing data on covariates and utilizing multiple imputation for missing covariates (Supplemental Tables 6 and 7). Exclusion of participants that occurred in the first 180 days of follow-up and applying competing risk models did not largely alter the estimates (Supplemental Tables 8 and 9). The association between TDI and progression to CVD, T2D, CKD, and CRMM was weaker than the main results but still significant (Supplemental Table 10). The analysis revealed a statistically significant association between SES and TDI categories, as shown in Supplemental Table 11 (LCA-defined SES: Cramér's V = 0.140, *P* < 0.001; summed SES score: Cramer's V = 0.090, *P* < 0.001). When the TDI was simultaneously included in the final model, the associations of individual level SES with primary outcomes were not materially changed (Supplemental Table 12). Stratified analyses by TDI levels demonstrated that the association between low SES and CRMDs was consistent across all TDI levels, indicating the robustness of our primary findings. Detailed results are provided in Supplemental Tables 13 and 14. In addition, in the context of transition pattern A, we employed varying time intervals for patients entering different states at the same time, and the findings remained robust and consistent (Supplemental Tables 15 and 16).

## Discussion

In this large-scale cohort study of 394,208 adults, low SES, whether defined by LCA or summed score, was independently associated with an elevated risk of CRMD, aligning with existing epidemiological evidence.^40^, ^41^, ^42^, ^43^, ^44^, ^45^ Contemporary studies reported associations between higher SES and reduced risks of CVD (HR: 0.87; 95% CI: 0.82-0.93), T2D (HR: 0.79; 95% CI: 0.75-0.84), and all-cause mortality (HR: 0.84; 95% CI: 0.81-0.88), reflecting systemic socioeconomic gradients in chronic disease.^41^ These associations were in accordance with population-based studies in diverse settings. For instance, a prior cohort analysis of the UK Biobank dataset (n = 122,494, age ≥60 years) revealed that participants with lower SES had a 10% increased risk of incident atrial fibrillation compared to those with high-SES.^44^ Moreover, low SES significantly predicted incident T2D and accelerated progression toward kidney failure and advanced cardiorenal metabolic stages.^46^, ^47^, ^48^, ^49^, ^50^, ^51^, ^52^, ^53^, ^54^

The socioeconomic disparities in the incidence of single cardiometabolic diseases have been well documented. Our findings further demonstrated that low SES is strongly associated with an elevated risk of CRMM. Considering that the relationships may be obscured by the aggregated multimorbidity indicators, we conducted a pattern-specific analysis of multimorbidity (e.g., CVD+T2D, T2D+CKD) across socioeconomic strata. Individuals with low SES had a significantly increased risk of developing these distinct comorbidities. The coexistence of multiple conditions creates a pathophysiological interplay that not only amplifies the physiological burden on individuals but also significantly increases the risk of complications, leading to higher rates of disability and mortality.^55^^,^^56^ These observational findings expose critical limitations of single-disease prevention frameworks, underscoring the imperative for public health strategies that target pathophysiological synergies among cardiorenal metabolic conditions, particularly in socioeconomically underserved populations.

Consistent with our findings, Mónica et al.^43^ confirmed a heightened CVD susceptibility in populations with low income and limited educational attainment. In addition, a Dutch registry analysis of 15,000 hospitalized patients with acute myocardial infarction or coronary heart disease demonstrated significantly elevated 28-day and 1-year mortality rates in the lowest income quintile group.^45^ Similar results were also reported in Asia-based studies.^46^^,^^47^ Lower income directly constrains access to and affordability of health care, limiting utilization of preventive services, guideline-concordant treatments, and long-term medication adherence.^57^ Concurrently, financial hardship increases the likelihood of exposure to adverse environmental and behavioral conditions, such as air pollution, and unhealthy dietary patterns.^58^ Educational attainment primarily influences health outcomes through health literacy and associated behaviors. Individuals with limited education may have a diminished capacity to understand health information, communicate effectively with providers, and appreciate the importance of risk factor management,^59^ leading to poorer self-care and underutilization of health care resources. Unemployment is a potent psychosocial stressor, which is linked to a higher risk of acute myocardial infarction.^60^ This increased risk is likely mediated by the cumulative psychological stress, loss of social identity, and disruption of health insurance associated with job loss. In summary, low SES confers a substantially increased risk for cardiorenal metabolic conditions through the synergistic interplay of material deprivation, limited health literacy, and chronic psychosocial stress.

SES demonstrated significant associations across all stages of disease progression, from free of CRMD to first CRMD, to CRMM, and ultimately to death. These results are consistent with evidence from the Whitehall II cohort studies.^55^ By further dividing first CRMD into three CRMDs, our study indicated that low SES independently increased the risk of progression from CVD or T2D to CRMM and all-cause mortality, consistent with emerging evidence on socioeconomic disparities in longitudinal multimorbidity progression.^61^ These findings underscore SES as a critical modifier of disease progression across divergent pathological pathways. This phenomenon can be explained by several factors. Low SES populations face barriers to health care access, resulting in delayed diagnosis and management of chronic conditions such as hypertension, diabetes, and hyperlipidemia, increasing the risk of noncommunicable diseases.^62^, ^63^, ^64^, ^65^, ^66^ They may also interrupt treatment due to inability to afford medication, resulting in a compounded disease burden.^57^, ^58^, ^59^, ^60^ Concurrently, these individuals exhibit higher prevalence of adverse health behaviors, such as smoking, alcohol consumption, and poor dietary patterns, which are well-established risk factors for mortality.^67^^,^^68^ However, low SES (LCA-defined) did not significantly influence progression from CKD to CRMM or mortality following CRMM, likely attributable to the limited number of transition events observed for CKD to CRMM (n = 343) and CRMD to mortality (n = 614) in our cohort.

Through multistate modeling, we identified a statistically significant socioeconomic gradient, demonstrating that SES not only influences progression from baseline to first CRMD but also serves as a robust predictor of subsequent transitions from first CRMD to CRMM and mortality. Cardiovascular, renal, and metabolic diseases frequently overlap and co-occur within individuals, sharing common pathophysiological mechanisms and reciprocal risk factors.^4^ Individuals with a single CRMD demonstrate heightened susceptibility to developing comorbid conditions. CRMDs share fundamental dysregulations, including endothelial dysfunction, atherogenesis, thrombosis, myocardial injury, fibrosis, and cardiac remodeling.^6^ For instance, insulin resistance and hyperglycemia in T2D accelerate atherosclerosis in CVD and promote renal fibrosis in CKD.^69^^,^^70^ Conversely, The hallmarks of CKD, albuminuria, and low glomerular filtration rate are associated with progressive increases in the risk of major atherosclerotic vascular and heart failure events and cardiovascular death.^71^ These shared pathways create a milieu where the presence of 1 CRMD significantly lowers the threshold for developing others and amplifies end-organ damage, explaining the disproportionate increase in morbidity and mortality observed in CRMM beyond the additive risks of the individual conditions.

Another key finding of this study is the marked heterogeneity in the impact of low SES on disease progression. Individuals with pre-existing CVD or T2D from disadvantaged backgrounds faced a significantly elevated risk of developing CRMM. The management of CVD and T2D relies heavily on sustained self-care, medication adherence, and regular follow-up—all of which are disproportionately impaired low-SES populations.^72^^,^^73^ In contrast, the association between SES and progression from CKD to CRMM was weak and nonsignificant, potentially due to limited statistical power from the smaller number of CKD progression events. These findings support the social inequality of CRMM progression and socioeconomic factors should be targeted for the early risk stratification and prevention of these diseases. For individuals with established CRMD from low-SES backgrounds, intensified secondary prevention strategies should be implemented, including medication cost subsidies and structured disease management education.

### Strengths

This study leverages the large sample size and long-term follow-up of the UK Biobank to capture the impact of SES on CRMM, employing Cox proportional hazards models and competing risks models. Concurrently, we characterized the influence of SES on the development into different multimorbidity patterns and confirmed the cumulative effect of the disease. By implementing multistate modeling, we characterized the longitudinal associations between SES and CRMM progression, uncovering significant socioeconomic gradients across all transition paths. Second, we constructed SES variable by LCA and the summed score base on total household income, educational qualifications, and employment status, revealing the association between SES and CRMM progression was robust. Using the area-level SES index (TDI) also supported our findings.

### Study Limitations

Our study also has several limitations. First, SES was assessed solely at baseline, failing to account for possible changes during the follow-up. Such dynamic changes could influence the observed associations, and future studies with repeated measures of SES are warranted to explore this aspect.^74^ Second, socioeconomic and lifestyle factors in the UK Biobank were predominantly self-reported, which may be subject to recall bias and social desirability bias. Third, our study only focused on the middle-aged and older adults in the United Kingdom, which may limit generalizability to populations with younger age and from other regions. Fourth, disease ascertainment relied on electronic health records, resulting in underascertainment of milder cases not meeting hospitalization criteria. Fifth, in the multistate models, participants entering multiple states on the same date were assigned a 0.5-day interval to prioritize transitions, a method which potentially introduces misclassification. However, sensitivity analyses using alternative intervals (30, 180, and 365 days) yielded consistent results. Finally, although we adjusted for key confounders, residual confounding from unmeasured factors cannot be entirely excluded.

In conclusion, SES functions not merely as a correlate factor but as an amplifier of CRMM. Socioeconomically disadvantaged individuals exhibit disproportionately elevated CRMM risk, where pathophysiological synergies among the diseases amplify multimorbidity and mortality beyond the summed impact of individual diseases. The longitudinal association between low SES and disease progression, from initial CRMD to CRMM and ultimately mortality, reveals a significant socioeconomic gradient in the development of CRMM. These findings suggest the importance of screening for SES in the populations for the prevention and treatment of CRMM. Critically, clinical resource allocation must prioritize SES vulnerability assessments, coupled with precision public health interventions targeting populations bearing the highest cumulative disease burdens: specifically, individuals characterized by low income, limited educational attainment, and unemployment status. Integrating SES into public health care strategies is imperative to address the systemic drivers of CRMM and mitigate health inequities across disease progression stages.

Perspectives **COMPETENCY IN MEDICAL KNOWLEDGE:** Integrating low SES into routine health assessments is essential, as it is a pivotal driver of CRMM risk and allows for the precise identification of individuals at elevated risk.

**TRANSLATIONAL OUTLOOK:** The priority of public health should be to develop and evaluate interventions that tackle socioeconomic barriers, breaking the link between low SES and the progression to multimorbidity and high mortality.

## Funding support and author disclosures

Dr Xu was supported by the 10.13039/100001547China Medical Board (21-416), the 10.13039/501100001809Natural Science Foundation of China (72474197), 10.13039/501100002771Zhejiang University, 10.13039/501100012226Fundamental Research Funds for the Central Universities, and the Key Laboratory of Intelligent Preventive Medicine of Zhejiang Province during the conduction of the study. The funders of the study had no role in study design, data collection, data analysis, data interpretation, or writing of the report. All other authors have reported that they have no relationships relevant to the contents of this paper to disclose.
