# Supplementary material for: Associations of Socioeconomic Status With Cardiorenal Metabolic Multimorbidity: Evidence From the UK Biobank Cohort
Source: JACC Adv. 2026 Mar 13;5(4):102646. doi: 10.1016/j.jacadv.2026.102646 (PMC12995894; doi:10.1016/j.jacadv.2026.102646)
Supplement: Supplemental_Material [file mmc1.pdf]

Supplementary Material

Supplementary Method..**1**

Text S1. The details of the socioeconomic status assessment..................................1

Text S2. The details of the lifestyle factors assessment 9

Text S3. The definitions of the diseases..11

Supplementary Tables..**12**

Table S1. Comparison of characteristics of participants who were excluded in the analyses versus included individuals.. 12

Table S2. Baseline characteristics of study participants according to summed socioeconomic status score...15

Table S3. Associations of different patterns of socioeconomic status with cardio-renal-metabolic diseases (n=394,208)...18

Table S4. Associations of different patterns of socioeconomic status with patterns of cardio-renal metabolic multimorbidity (n=394,208)...19

Table S5. Associations of income, education and employment status with cardio-renal-metabolic diseases (n=394,208)...20

Table S6. Associations of different patterns of socioeconomic status with cardio-renal-metabolic diseases by excluding individuals with missing information on covariates (n=323,536)... 21

Table S7. Associations of different patterns of socioeconomic status with cardio-renal-metabolic diseases by imputing missing covariates with multiple imputation (n=394,208).. 22

Table S8. Associations of different patterns of socioeconomic status with cardio-renal-metabolic diseases by excluding outcome events that occurred in the first 180 days of follow-up (n=392,737)..23

Table S9. Associations of different patterns of socioeconomic status with cardio-renal-metabolic diseases using the competing risk model (n = 394,208)..... 24

Table S10. Associations of Townsend deprivation index with cardio-renal-metabolic diseases (n=394,208).. 24

Table S11. Correlation between different patterns of socioeconomic status and Townsend deprivation index (n = 394,208)... 25

Table S12. Associations of different patterns of socioeconomic status with cardio-renal-metabolic diseases after adjusting Townsend deprivation index (n = 394,208).. 25

Table S13. Associations of patterns of socioeconomic status defined by latent class analysis with cardio-renal-metabolic diseases, stratified by Townsend deprivation Index (n = 394,208).. 26

Table S14. Associations of patterns of summed socioeconomic status score with cardio-renal-metabolic diseases, stratified by Townsend deprivation index (n = 394,208).. 26

Table S15. Association of socioeconomic status defined by latent class analysis with the disease progression, using different intervals for the participants entering different states on the same date (n=394,208).. 27

Table S16. Association of summed socioeconomic status score with the disease progression, using different intervals for the participants entering different states on the same date (n=394,208)... 28

Supplementary Figures.. **29**

Figure S1. Flow chart of the selection process 29

Figure S2. The Kaplan-Meier curve for different outcomes across socioeconomic status defined by latent class analysis... 30

Figure S3. The Kaplan-Meier curve for different outcomes across summed socioeconomic status score... 30

Supplementary Method

Text S1. The details of the socioeconomic status assessment

As mentioned in an earlier study[1,2], socioeconomic status (SES) the following three factors: total household income level before tax, education qualifications and employment status. All information on SES factors was measured at baseline using a touchscreen questionnaire. Detailed information can be obtained from the following table.

Table for supplementary method. Definitions of total household income level before tax, education qualifications and employment status.

Subvariables	UK Biobank field code	Definition	Note
Total household income level before tax	738	Grouped into 5 levels:<br>(i) < £18,000;<br>(ii) £18,000–£30,999;<br>(iii) £31,000–£51,999;<br>(iv) £52,000–£100,000;<br>(v) > £100,000	

Education qualifications	6138	Grouped into 7 levels:<br>(i) College or University degree;<br>(ii) A levels, AS levels, or equivalent;<br>(iii) O levels, GCSEs, or equivalent;<br>(iv) CSEs or equivalent;<br>(v) NVQ, HND, HNC, or equivalent;<br>(vi) Other professional qualifications eg: nursing, teaching;<br>(vii) Equivalent to or less than high school diploma	" Equivalent to or less than high school diploma" was defined as "none of the above"
Employment status	6142	Employed status was defined as meeting one of the following:<br>(i) in paid employment or self-employed;<br>(ii) retired;<br>(iii) doing unpaid or voluntary work;<br>(iv) full or part-time student	

We evaluated latent class analysis (LCA) models with 2-10 potential classes. Models failed to converge when specifying more than 4 classes. Consequently,we only reported information on models with four or fewer latent classes. Subsequently, we employed a multi-criteria decision framework incorporating the Akaike Information Criterion (AIC), Bayesian Information Criterion (BIC), and likelihood ratio statistics (G^2) for optimal class selection, retaining latent classes with mean posterior probabilities exceeding 0.7 as classifications. Based on the item response probabilities, we identified three distinct latent classes, corresponding to high, medium, and low SES levels.

Figure for supplementary method. G^2 statistics, AIC, and BIC in models with different numbers of latent classes.

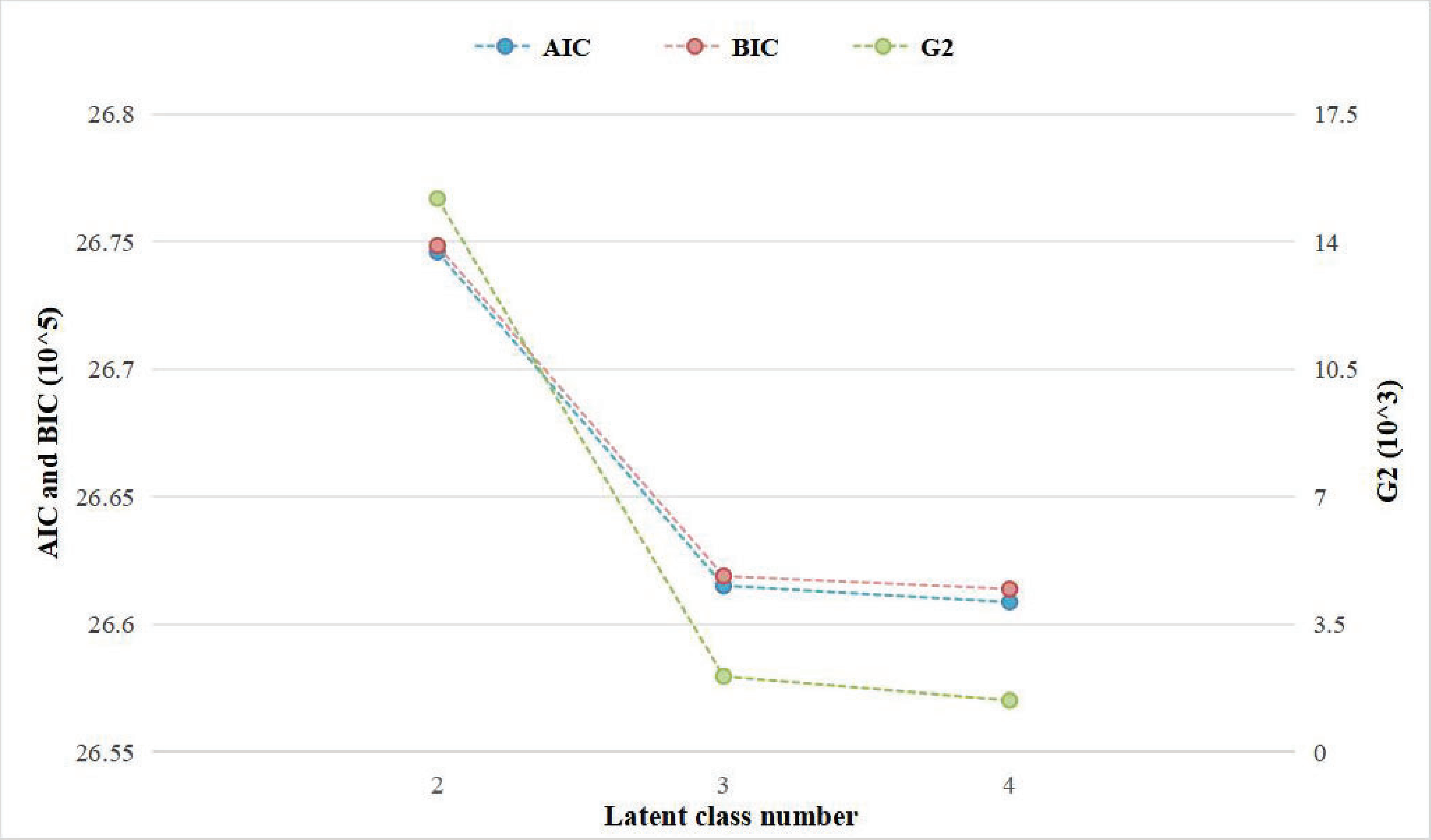

Table for supplementary method. Mean posterior probabilities, prevalence of latent classes, and item-response probabilities in models with two to four latent classes.

Item			Latent class 1	Latent class 2	Latent class 3	Latent class 4
Two-latent-class solution		MeanPP	0.88	0.86	——	——
		Prevalence	0.43	0.57	——	——
	Total household income level before tax (£)	> 100,000	0.00	0.10	——	——
		52,000–100,000	0.02	0.36	——	——
		31,000–51,999	0.15	0.36	——	——
		18,000–30,999	0.34	0.19	——	——
		< 18,000	0.50	0.00	——	——
	Education qualifications	College or University degree	0.14	0.52	——	——
		A levels/AS levels or equivalent	0.09	0.14	——	——
		O levels/GCSEs or equivalent	0.25	0.19	——	——
		CSEs or equivalent	0.07	0.04	——	——
		NVQ or HND or HNC or equivalent	0.09	0.05	——	——
		Other professional qualifications	0.06	0.04	——	——
		None of the above	0.30	0.02	——	——
	Employment status	Employed	0.88	0.97	——	——
		Unemployed	0.12	0.03	——	——
Three-latent-class solution		MeanPP	**0.77**	**0.80**	**0.86**	——
		Prevalence	0.47	0.23	0.30	——
	Total	> 100,000	0.00	0.23	0.01	——

	household income level before tax (£)	52,000–100,000	0.18	**0.54**	0.01	——
		31,000–51,999	**0.43**	0.23	0.04	——
		18,000–30,999	0.40	0.00	0.23	——
		< 18,000	0.00	0.00	**0.71**	——
	Education qualifications	College or University degree	0.30	**0.73**	0.16	——
		A levels/AS levels or equivalent	0.14	0.13	0.08	——
		O levels/GCSEs or equivalent	**0.27**	0.09	0.22	——
		CSEs or equivalent	0.07	0.01	0.06	——
		NVQ or HND or HNC or equivalent	0.08	0.02	0.08	——
		Other professional qualifications	0.06	0.03	0.05	——
		None of the above	0.08	0.00	**0.35**	——
	Employment status	Employed	0.97	0.95	0.84	——
		Unemployed	0.03	0.05	**0.16**	——
Four-latent-class solution		MeanPP	0.85	1.00	0.55	0.74
		Prevalence	0.31	0.27	0.16	0.30
	Total household income level before tax (£)	> 100,000	0.14	0.01	0.07	0.00
		52,000–100,000	0.40	0.02	0.45	0.10
		31,000–51,999	0.31	0.03	0.47	0.35
		18,000–30,999	0.15	0.14	0.00	0.55
		< 18,000	0.00	0.80	0.00	0.00
	Education	College or University degree	0.89	0.16	0.02	0.11

qualifications	A levels/AS levels or equivalent	0.08	0.08	0.27	0.13
	O levels/GCSEs or equivalent	0.00	0.22	0.47	0.33
	CSEs or equivalent	0.00	0.06	0.09	0.09
	NVQ or HND or HNC or equivalent	0.00	0.08	0.10	0.10
	Other professional qualifications	0.03	0.05	0.05	0.07
	None of the above	0.00	0.34	0.00	0.16
Employment status	Employed	0.96	0.83	0.95	0.97
	Unemployed	0.04	0.17	0.05	0.03

Abbreviations: meanPP: mean posterior probability.

Figure for supplementary method. Definitions of high, medium, and low socioeconomic status in the three-latent-class solution.

	Employed					Unemployed				
	> £100,000	£52,000–£100,000	£31,000–£51,999	£18,000–£30,999	< £18,000	> £100,000	£52,000–£100,000	£31,000–£51,999	£18,000–£30,999	< £18,000
College or University degree	3.802	11.170	9.815	6.412	2.840	0.241	0.366	0.322	0.290	0.588
A levels/AS levels or equivalent	0.531	2.753	3.465	2.774	1.480	0.069	0.123	0.156	0.149	0.325
O levels/GCSEs or equivalent	0.447	3.355	5.937	6.134	3.988	0.059	0.152	0.259	0.332	0.741
CSEs or equivalent	0.062	0.684	1.625	1.544	1.008	0.010	0.036	0.076	0.121	0.333
NVQ or HND or HNC or equivalent	0.114	0.901	1.670	1.878	1.476	0.007	0.021	0.047	0.083	0.262
Other professional qualifications	0.153	0.830	1.306	1.500	0.963	0.013	0.025	0.040	0.051	0.123
None of the above	0.088	0.575	1.780	3.727	6.160	0.011	0.051	0.113	0.263	1.190

The numbers in the cells represented the percentage of participants out of the total study population.

20.257	High	53.023	Medium	26.715	Low

We developed an alternative socioeconomic status (SES) assessment protocol by constructing a composite summed SES score based on three validated indicators (total household income level before tax, education qualifications and employment status), following methodological precedents from prior studies[3]. Scores were summed across dimensions (range: 0-3) and operationalized as three discrete strata: low [0], medium [1-2], and high [3].

Table for supplementary method. Details of the summed socioeconomic status score.

Subvariables	Definition
Total household income level before tax	"< £18,000", £18,000–£30,999 and "£31,000–£51,999" were scored as 0.
	"£52,000–£100,000" and "> £100,000" were scored as 1.
Education qualifications	"None of the above", "CSEs or equivalent", "NVQ, HND, HNC, or equivalent", "Other professional qualifications eg: nursing, teaching", “A levels, AS levels, or equivalent” and "O levels, GCSEs, or equivalent" were scored as 0.
	“College or University degree” was scored as 1.
Employment status	"Looking after home and/or family", "unable to work because of sickness or disability" and "unemployed" were scored as 0.
	"In paid employment or self-employed", "retired", "doing unpaid or voluntary work" and "full or part-time student" were scored as 1.

Figure for supplementary method. Definitions of high, medium, and low socioeconomic status in the summed scoring framework.

	Employed					Unemployed				
	> £100,000	£52,000–£100,000	£31,000–£51,999	£18,000–£30,999	< £18,000	> £100,000	£52,000–£100,000	£31,000–£51,999	£18,000–£30,999	< £18,000
College or University degree	3.802	11.170	9.815	6.412	2.840	0.241	0.366	0.322	0.290	0.588
A levels/AS levels or equivalent	0.531	2.753	3.465	2.774	1.480	0.069	0.123	0.156	0.149	0.325
O levels/GCSEs or equivalent	0.447	3.355	5.937	6.134	3.988	0.059	0.152	0.259	0.332	0.741
CSEs or equivalent	0.062	0.684	1.625	1.544	1.008	0.010	0.036	0.076	0.121	0.333
NVQ or HND or HNC or equivalent	0.114	0.901	1.670	1.878	1.476	0.007	0.021	0.047	0.083	0.262
Other professional qualifications	0.153	0.830	1.306	1.500	0.963	0.013	0.025	0.040	0.051	0.123
None of the above	0.088	0.575	1.780	3.727	6.160	0.011	0.051	0.113	0.263	1.190

The numbers in the cells represented the percentage of participants out of the total study population.

14.972	High [3]	80.359	Medium [1-2]	4.664	Low [0]

Text S2. The details of the lifestyle factors assessment

For lifestyle factors, we selected five variables for assessment: healthy diet, smoking status, drinking status, physical activity, and sleep duration. The specific definitions are as follows:

Table for supplementary method. Definitions of lifestyle factors.

Variables	UK Biobank field code	Definition	Note
Healthy diet[4]	1309, 1319, 1289, 1299, 1329, 1339, 1349, 1369, 1379, 1389, 1438, 1448, 1458, 1468	Meet at least 4 of the following 7 patterns:<br>(i) Fruits >= 3servings/day;<br>(ii) Vegetables >= 3 servings/day;<br>(iii) Fish >= 2 servings/week;<br>(iv) Processed meats < = 1 servings/week;<br>(v) Unprocessed red meats < = 1.5 servings/week;<br>(vi) Whole grains > = 3 servings/day;<br>(vii) Refined grains: <=1.5 servings/day	A healthy dietary pattern was scored as 1.
Smoking status	20116	Grouped into 4 levels:<br>(i) Never smoking;<br>(ii) Previous smoking;<br>(iii) Current smoking;<br>(iv) Prefer not to answer.	"Prefer not to answer" was defined as "unknown"

Drinking status	20117	Grouped into 4 levels:<br>(i) Never drinking;<br>(ii) Previous drinking;<br>(iii) Current drinking;<br>(iv) Prefer not to answer.	" Prefer not to answer" was defined as "unknown"
Physical activity	22032	Grouped into 4 levels:<br>(i) Low;<br>(ii) Moderate;<br>(iii) High;<br>(iv) Missing values.	"Missing values" was defined as "unknown"
Sleep duration[5]	1160	Grouped into 4 levels:<br>(i) <7h;<br>(ii) 7-9h;<br>(iii) >9h;<br>(iv) Prefer not to answer or do not know.	"Prefer not to answer or do not know" was defined as "unknown"

Text S3. The definitions of the diseases

Cardio-renal-metabolic multimorbidity (CRMM) was defined as the coexistence of two or three cardio-renal-metabolic diseases (CRMDs), including CVD, T2D and CKD. CVD was defined using codes corresponding to coronary heart disease, atrial fibrillation, heart failure, peripheral artery disease and stroke. The corresponding ICD-10 codes are tabulated below.

Table for supplementary method. Definitions of CRMM, COPD and cancer.

Variables	Subvariables	UK Biobank field code	Definition
CRMM	CVD	41270	ICD-10: I11, I20-I25, I42.1, I42.5, I42.8, I42.9,I48-I50, I64, I70.0, I70.2, I70.8, I70.9, I73.8, I73.9.
	CKD		ICD-10: N18
	T2D		ICD-10: E11, E14
COPD			ICD-10: J440, J441, J448, J449
Cancer			ICD-10: C00 - C97, D00 - D09

Abbreviations: CVD, Cardiovascular diseases; T2D, Type 2 diabetes; CKD, Chronic kidney disease; COPD, Chronic obstructive pulmonary disease.

Supplementary Tables

Table S1. Comparison of characteristics of participants who were excluded in the analyses versus included individuals.

Characteristics	Overall (n=502 490)	Excluded (n=108 282)	Included (n=394,208)	*P*
**Age (year, mean, [SD])**	56.5 (8.1)	59.1 (7.6)	55.8 (8.1)	<0.001
**Sex (n, %)**				<0.001
Female	273,375 (54.4)	60,284 (55.7)	213,091 (54.1)	
Male	229,114 (45.6)	47,997 (44.3)	181,117 (45.9)	
**Ethnicity (n, %)**				<0.001
White	472,680 (94.1)	97,808 (90.3)	374,872 (95.1)	
Non-white	27,033 (5.4)	8,693 (8.0)	18,340 (4.7)	
Unknown	2,777 (0.6)	1,781 (1.6)	996 (0.3)	
**Total household income level before tax (n, %)**				<0.001
> £100,000	22,929 (5.4)	823 (2.6)	22,106 (5.6)	
£52,000–£100,000	86,262 (20.3)	3,311 (10.6)	82,951 (21.0)	
£31,000–£51,999	110,770 (26.0)	5,869 (18.9)	104,901 (26.6)	
£18,000–£30,999	108,174 (25.4)	8,589 (27.6)	99,585 (25.3)	
< £18,000	97,194 (22.9)	12,529 (40.3)	84,665 (21.5)	
**Education qualification (n, %)**				<0.001
College or University degree	161,158 (32.7)	19,848 (20.2)	141,310 (35.8)	
A levels/AS levels or equivalent	55,321 (11.2)	8,709 (8.9)	46,612 (11.8)	
O levels/GCSEs or equivalent	105,194 (21.4)	20,810 (21.2)	84,384 (21.4)	
CSEs or equivalent	26,887 (5.5)	5,205 (5.3)	21,682 (5.5)	
NVQ or HND or HNC or equivalent	32,726 (6.7)	7,265 (7.4)	25,461 (6.5)	
Other professional qualifications	25,802 (5.2)	6,069 (6.2)	19,733 (5.0)	
None of the above	85,269 (17.3)	30,243 (30.8)	55,026 (14.0)	
**Employment status (n, %)**				<0.001
Employed	457,787 (91.6)	91,362 (86.7)	366,425 (93.0)	
Unemployed	41,751 (8.4)	13,968 (13.3)	27,783 (7.0)	
**TDI (n, %)**				<0.001
Low	167,374 (33.4)	33,216 (30.9)	132,907 (33.7)	
Medium	167,204 (33.3)	34,404 (32.0)	131,880 (33.5)	
High	167,288 (33.3)	40,038 (37.2)	129,421 (32.8)	
**BMI (n, %)**				<0.001
<18.5 kg/m^2	5,731 (1.1)	1,994 (1.8)	3,737 (0.9)	
18.5-24.9 kg/m^2	157,420 (31.3)	28,858 (26.7)	128,562 (32.6)	
25-29.9 kg/m^2	209,225 (41.6)	43,463 (40.1)	165,762 (42.1)	

>=30 kg/m^2	122,243 (24.3)	32,325 (29.9)	89,918 (22.8)	
Unknown	7,871 (1.6)	1,642 (1.5)	6,229 (1.6)	
**Healthy diet (n, %)**				<0.001
Yes	224,942 (44.8)	46,114 (42.6)	178,828 (45.4)	
No	277,548 (55.2)	62,168 (57.4)	215,380 (54.6)	
**Smoking status (n, %)**				<0.001
Never	273,514 (54.4)	55,524 (51.3)	217,990 (55.3)	
Previous	173,050 (34.4)	39,014 (36)	134,036 (34.0)	
Current	52,977 (10.5)	11,789 (10.9)	41,118 (10.4)	
Unknown	2,949 (0.6)	1,955 (1.8)	994 (0.3)	
**Drinking status (n, %)**				<0.001
Never	22,385 (4.5)	7,805 (7.2)	14,580 (3.7)	
Previous	18,102 (3.6)	5,448 (5.0)	12,654 (3.2)	
Current	460,349 (91.6)	93,660 (86.5)	366,689 (93.0)	
Unknown	1,654 (0.3)	1,369 (1.3)	285 (0.1)	
**Physical activity (n, %)**				<0.001
Low	76,215 (15.2)	14,896 (13.8)	61,319 (15.6)	
Medium	164,017 (32.6)	28,323 (26.2)	135,694 (34.4)	
High	162,136 (32.3)	28,415 (26.2)	133,721 (33.9)	
Unknown	100,122 (19.9)	36,648 (33.8)	63,474 (16.1)	
**Sleep duration (n, %)**				<0.001
<7 h	123,246 (24.5)	28,761 (26.6)	94,485 (24.0)	
7-9 h	365,786 (72.8)	73,488 (67.9)	292,298 (74.1)	
>9 h	9,242 (1.8)	3,229 (3.0)	6,013 (1.5)	
Unknown	4,216 (0.8)	2,804 (2.6)	1,412 (0.4)	
**Hypertension (n, %)**				<0.001
Yes	235,845 (46.9)	53,376 (49.3)	182,469 (46.3)	
No	266,645 (53.1)	54,906 (50.7)	211,739 (53.7)	
**Use of antihypertensive medication (n, %)**				<0.001
Yes	104,793 (20.9)	36638 (33.8)	68,155 (17.3)	
No	397,697 (79.1)	71644 (66.2)	326,053 (82.7)	
**Dyslipidemia (n, %)**				<0.001
Yes	359,988 (71.6)	64,968 (60.0)	295,020 (74.8)	
No	142,502 (28.4)	43,314 (40.0)	99,188 (25.2)	
**Use of lipid-lowering medications (n, %)**				<0.001
Yes	90,883 (18.1)	37,965 (35.1)	52,918 (13.4)	
No	411,607 (81.9)	70,317 (64.9)	341,290 (86.6)	

**COPD (n, %)**				<0.001
Yes	13,355 (2.7)	5,692 (5.3)	7,663 (1.9)	
No	489,135 (97.3)	102,590 (94.7)	386,545 (98.1)	
**Cancer (n, %)**				<0.001
Yes	69,373 (13.8)	17,904 (16.5)	48,668 (12.3)	
No	433,117 (86.2)	90,378 (83.5)	345,540 (87.7)	
**CVD (n, %)**				<0.001
Yes	64,669 (12.9)	35,431 (32.7)	29,238 (7.4)	
No	437,821 (87.1)	72,851 (67.3)	364,970 (92.6)	
**T2D (n, %)**				<0.001
Yes	31,066 (6.2)	16,544 (15.3)	14,522 (3.7)	
No	471,424 (93.8)	91,738 (84.7)	379,686 (96.3)	
**CKD (n, %)**				<0.001
Yes	9,829 (2.0)	4,921 (4.5)	4,908 (1.3)	
No	492,661 (98.0)	103,361 (95.5)	389,300 (98.8)	
**Multimorbidity (n, %)**				
CVD and T2D	10,327 (2.1)	7,009 (6.5)	3,313 (0.8)	<0.001
CVD and CKD	3,020 (0.6)	1,677 (1.5)	1,343 (0.3)	<0.001
T2D and CKD	1,023 (0.2)	520 (0.5)	503 (0.1)	<0.001
CVD and T2D and CKD	2,189 (0.4)	1,705 (1.6)	484 (0.1)	<0.001

Abbreviations: SES: Socioeconomic status; TDI: Townsend deprivation index; BMI: Body mass index; COPD: Chronic obstructive pulmonary disease; CVD:Cardiovascular disease; T2D: Type 2 diabetes; CKD: Chronic kidney disease; CRMD: Cardio-renal-metabolic disease.

Table S2. Baseline characteristics of study participants according to summed socioeconomic status score.

Characteristics	Total (n=394,208)	High SES [3] (n=59,021)	Medium SES [1-2] (n=316,802)	Low SES [0] (n=18,385)
**Age (year, mean, [SD])**	55.8 (8.1)	52.6 (7.2)	56.6 (8.1)	53.3 (6.7)
**Sex (n, %)**				
Female	213,091 (54.1)	27,690 (46.9)	175,151 (55.3)	10,250 (55.8)
Male	181,117 (45.9)	31,331 (53.1)	141,651 (44.7)	8,135 (44.2)
**Ethnicity (n, %)**				
White	374,872 (95.1)	56,423 (95.6)	301,537 (95.2)	16,912 (92.0)
Non-white	18,340 (4.7)	2,483 (4.2)	14,462 (4.6)	1,395 (7.6)
Unknown	996 (0.3)	115 (0.2)	803 (0.2)	78 (0.4)
**Total household income level before tax (n, %)**				
> £100,000	22,106 (5.6)	14,988 (25.4)	7,118 (2.2)	0 (0)
£52,000–£100,000	82,951 (21.0)	44,033 (74.6)	38,918 (12.3)	0 (0)
£31,000–£51,999	104,901 (26.6)	0 (0)	102,181 (32.3)	2,720 (14.8)
£18,000–£30,999	99,585 (25.3)	0 (0)	95,642 (30.2)	3,943 (21.4)
< £18,000	84,665 (21.5)	0 (0)	72,943 (23.0)	11,722 (63.8)
**Education qualification (n, %)**				
College or University degree	141,310 (35.8)	59,021 (100.0)	82,289 (26.0)	0 (0)
A levels/AS levels or equivalent	46,612 (11.8)	0 (0)	44,132 (13.9)	2,480 (13.5)
O levels/GCSEs or equivalent	84,384 (21.4)	0 (0)	79,131 (25.0)	5,253 (28.6)
CSEs or equivalent	21,682 (5.5)	0 (0)	19,592 (6.2)	2,090 (11.4)
NVQ or HND or HNC or equivalent	25,461 (6.5)	0 (0)	23,917 (7.5)	1,544 (8.4)
Other professional qualifications	19,733 (5.0)	0 (0)	18,891 (6.0)	842 (4.6)
None of the above	55,026 (14.0)	0 (0)	48,850 (15.4)	6,176 (33.6)
**Employment status (n, %)**				
Employed	366,425 (93.0)	59,021 (100.0)	307,404 (97.0)	0 (0)
Unemployed	27,783 (7.0)	0 (0)	9,398 (3.0)	18,385 (100.0)
**TDI (n, %)**				
Low	132,907 (33.7)	23,747 (40.2)	105,797 (33.4)	3,363 (18.3)
Medium	131,880 (33.5)	19,340 (32.8)	107,999 (34.1)	4,541 (24.7)
High	129,421 (32.8)	15,934 (27.0)	103,006 (35.5)	10,481 (57.0)
**BMI (n, %)**				
<18.5 kg/m^2	3,737 (0.9)	454 (0.8)	2,868 (0.9)	415 (2.3)
18.5-24.9 kg/m^2	128,562 (32.6)	23,619 (40.0)	99,908 (31.5)	5,035 (27.4)
25-29.9 kg/m^2	165,762 (42.1)	24,482 (41.5)	134,697 (42.5)	6,583 (35.8)
>=30 kg/m^2	89,918 (22.8)	9,530 (16.1)	74,260 (23.4)	6,128 (33.3)

<18.5 kg/m²	6,229 (1.6)	936 (1.6)	5,069 (1.6)	224 (1.2)
**Healthy diet (n, %)**				
Yes	230,310 (58.4)	20,700 (48.6)	133,359 (55.1)	9,839 (65.7)
No	163,898 (41.6)	38,321 (51.4)	183,443 (44.9)	8,546 (34.3)
**Smoking status (n, %)**				
Never	217,990 (55.3)	37,347 (63.3)	172,582 (54.5)	8,061 (43.8)
Previous	134,036 (34.0)	18,021 (30.5)	110,409 (34.8)	5,606 (30.5)
Current	41,118 (10.4)	3,592 (6.1)	32,944 (10.4)	4,652 (25.3)
Unknown	994 (0.3)	61 (0.1)	867 (0.3)	66 (0.4)
**Drinking status (n, %)**				
Never	14,580 (3.7)	1,047 (1.8)	12,236 (3.9)	1,297 (7.1)
Previous	12,654 (3.2)	1,044 (1.8)	9,959 (3.1)	1,651 (9.0)
Current	366,689 (93.0)	56,913 (96.4)	294,399 (92.9)	15,377 (83.6)
Unknown	285 (0.1)	17 (0)*	208 (0.1)	60 (0.3)
**Physical activity (n, %)**				
Low	61,319 (15.6)	10,732 (18.2)	46,678 (14.7)	3,909 (21.3)
Medium	135,694 (34.4)	23,817 (40.4)	106,311 (33.6)	5,566 (30.3)
High	13,3721 (33.9)	18,948 (32.1)	109,873 (34.7)	4,900 (26.6)
Unknown	63,474 (16.1)	5,524 (9.4)	53,940 (17.0)	4,010 (21.8)
**Sleep duration (n, %)**				
<7 h	94,485 (24.0)	12,448 (21.1)	76,239 (24.1)	5,798 (31.5)
7-9 h	292,298 (74.1)	46,328 (78.5)	234,848 (74.1)	11,122 (60.5)
>9 h	6,013 (1.5)	220 (0.4)	4,626 (1.5)	1,167 (6.4)
Unknown	1,412 (0.4)	25 (0)*	1,089 (0.3)	298 (1.6)
**Hypertension (n, %)**				
Yes	182,469 (46.3)	22,652 (38.4)	151,611 (47.9)	8,206 (44.6)
No	211,739 (53.7)	36,369 (61.6)	165,191 (52.1)	10,179 (55.4)
**Use of antihypertensive medication (n, %)**				
Yes	68,155 (17.3)	6,280 (10.6)	58,055 (18.3)	3,820 (20.8)
No	326,053 (82.7)	52,741 (89.4)	258,747 (81.7)	14,565 (79.2)
**Dyslipidemia (n, %)**				
Yes	295,020 (74.8)	44,094 (74.7)	237,605 (75.0)	13,321 (72.5)
No	99,188 (25.2)	14,927 (25.3)	79,197 (25.0)	5,064 (27.5)
**Use of lipid-lowering medications (n, %)**				
Yes	52,918 (13.4)	5,092 (8.6)	44,664 (14.1)	3,162 (17.2)
No	341,290 (86.6)	53,929 (91.4)	272,138 (85.9)	15,223 (82.8)
**COPD at baseline (n, %)**				

Yes	7,663 (1.9)	217 (0.4)	6,301 (2.0)	1,145 (6.2)
No	386,545 (98.1)	58,804 (99.6)	310,501 (98.0)	17,240 (93.8)
**Cancer at baseline (n, %)**				
Yes	51,469 (13.1)	5,670 (9.0)	43,317 (12.9)	2,482 (12.8)
No	342,739 (86.9)	53,351 (91.0)	273,485 (87.1)	15,903 (87.2)
**CVD (n, %)**				
Yes	29,238 (7.4)	2,653 (4.5)	24,703 (7.8)	1,882 (10.2)
No	364,970 (92.6)	56,368 (95.5)	292,099 (92.2)	16,503 (89.8)
**T2D (n, %)**				
Yes	14,522 (3.7)	994 (1.7)	12,117 (3.8)	1,411 (7.7)
No	379,686 (96.3)	58,027 (98.3)	304,685 (96.2)	16,974 (92.3)
**CKD (n, %)**				
Yes	4,908 (1.3)	282 (0.5)	4,291 (1.4)	335 (1.8)
No	389,300 (98.8)	58,739 (99.5)	312,511 (98.6)	18,050 (98.2)
**Multimorbidity (n, %)**				
CVD and T2D	3,313 (0.8)	181 (0.3)	2,774 (0.9)	358 (2.0)
CVD and CKD	1,343 (0.3)	62 (0.1)	1,196 (0.4)	85 (0.5)
T2D and CKD	503 (0.1)	23 (0)*	449 (0.1)	31 (0.2)
CVD and T2D and CKD	484 (0.1)	15 (0)*	424 (0.1)	45 (0.2)

* The value is <0.1% but rounds to 0.0% when formatted to one decimal place.

P values were calculated using analysis of variance and χ^2 test for continuous and categorical variables, respectively. All P values were <0.001.

Abbreviations: SES: Socioeconomic status; TDI: Townsend deprivation index; BMI: Body mass index; COPD: Chronic obstructive pulmonary disease; CVD:Cardiovascular disease; T2D: Type 2 diabetes; CKD: Chronic kidney disease; CRMD: Cardio-renal-metabolic disease.

Table S3. Associations of different patterns of socioeconomic status with cardio-renal-metabolic diseases (n=394,208).

		HR (95% CI)			
		**Model 1**	**Model 2**	**Model 3**	**Model 4**
**CVD**					
LCA-defined SES	High	1 (Ref)	1 (Ref)	1 (Ref)	1 (Ref)
	Medium	1.55 (1.50, 1.61)	1.25 (1.20, 1.29)	1.17 (1.13, 1.21)	1.14 (1.10, 1.19)
	Low	2.58 (2.48, 2.68)	1.69 (1.63, 1.76)	1.43 (1.38, 1.49)	1.31 (1.26, 1.37)
Summed SES score	High [3]	1 (Ref)	1 (Ref)	1 (Ref)	1 (Ref)
	Medium [1-2]	1.78 (1.71, 1.85)	1.34 (1.29, 1.39)	1.21 (1.16, 1.26)	1.17 (1.12, 1.22)
	Low [0]	2.39 (2.26, 2.54)	2.49 (2.35, 2.64)	1.87 (1.76, 1.99)	1.62 (1.52, 1.72)
**T2D**					
LCA-defined SES	High	1 (Ref)	1 (Ref)	1 (Ref)	1 (Ref)
	Medium	1.83 (1.73, 1.94)	1.61 (1.52, 1.70)	1.40 (1.32, 1.48)	1.35 (1.28, 1.43)
	Low	3.63 (3.43, 3.85)	2.75 (2.59, 2.92)	1.97 (1.86, 2.10)	1.73 (1.62, 1.83)
Summed SES score	High [3]	1 (Ref)	1 (Ref)	1 (Ref)	1 (Ref)
	Medium [1-2]	2.31 (2.17, 2.47)	1.89 (1.77, 2.02)	1.53 (1.43, 1.64)	1.46 (1.37, 1.56)
	Low [0]	4.80 (4.43, 5.21)	4.74 (4.37, 5.14)	2.74 (2.52, 2.98)	2.18 (2.00, 2.37)
**CKD**					
LCA-defined SES	High	1 (Ref)	1 (Ref)	1 (Ref)	1 (Ref)
	Medium	2.36 (2.11, 2.64)	1.69 (1.51, 1.89)	1.55 (1.39, 1.73)	1.49 (1.33, 1.66)
	Low	4.98 (4.46, 5.56)	2.61 (2.33, 2.93)	2.08 (1.85, 2.33)	1.82 (1.62, 2.05)
Summed SES score	High [3]	1 (Ref)	1 (Ref)	1 (Ref)	1 (Ref)
	Medium [1-2]	2.88 (2.55, 3.24)	1.80 (1.59, 2.03)	1.56 (1.38, 1.76)	1.47 (1.30, 1.66)
	Low [0]	3.95 (3.37, 4.63)	3.91 (3.34, 4.58)	2.62 (2.23, 3.09)	2.11 (1.79, 2.48)
**CRMM**					
LCA-defined SES	High	1 (Ref)	1 (Ref)	1 (Ref)	1 (Ref)
	Medium	2.38 (2.14, 2.65)	1.84 (1.65, 2.04)	1.62 (1.46, 1.81)	1.55 (1.40, 1.73)
	Low	5.58 (5.03, 6.21)	3.37 (3.02, 3.76)	2.41 (2.15, 2.69)	2.03 (1.82, 2.27)
Summed SES score	High [3]	1 (Ref)	1 (Ref)	1 (Ref)	1 (Ref)
	Medium [1-2]	3.25 (2.89, 3.67)	2.25 (2.00, 2.54)	1.83 (1.62, 2.07)	1.71 (1.51, 1.93)
	Low [0]	6.15 (5.32, 7.11)	6.34 (5.48, 7.33)	3.49 (3.00, 4.04)	2.60 (2.24, 3.02)

Model 1: unadjusted.

Model 2: adjusted for age at baseline, sex and ethnicity.

Model 3: model 2 also adjusted for BMI at baseline, current smoking and drinking status, healthy diet, sleep duration and physical activity.

Model 4: model 3 also adjusted for hypertension, dyslipidemia, medical history, COPD and cancer at baseline.

Abbreviations: SES, Socioeconomic status; LCA, Latent class analysis; HR, Hazard ratio; CI, Confidence interval; CVD, Cardiovascular diseases; T2D, Type 2 diabetes; CKD, Chronic kidney disease; CRMM, Cardio-renal-metabolic multimorbidity .

All P value are statistically significant (P < 0.001).

Table S4. Associations of different patterns of socioeconomic status with patterns of cardio-renal metabolic multimorbidity (n=394,208).

		HR (95% CI)			
		**Model 1**	**Model 2**	**Model 3**	**Model 4**
**CVD and T2D**					
LCA-defined SES	High	1 (Ref)	1 (Ref)	1 (Ref)	1 (Ref)
	Medium	2.17 (1.90, 2.48)	1.80 (1.57, 2.06)	1.58 (1.38, 1.81)	1.51 (1.32, 1.73)
	Low	4.94 (4.33, 5.63)	3.41 (2.98, 3.91)	2.40 (2.09, 2.76)	2.03 (1.77, 2.33)
Summed SES score	High [3]	1 (Ref)	1 (Ref)	1 (Ref)	1 (Ref)
	Medium [1-2]	2.89 (2.49, 3.36)	2.20 (1.89, 2.56)	1.77 (1.52, 2.06)	1.66 (1.43, 1.94)
	Low [0]	6.56 (5.49, 7.84)	6.77 (5.66, 8.10)	3.65 (3.04, 4.38)	2.73 (2.27, 3.28)
**CVD and CKD**					
LCA-defined SES	High	1 (Ref)	1 (Ref)	1 (Ref)	1 (Ref)
	Medium	2.60 (2.08, 3.25)	1.71 (1.37, 2.14)	1.57 (1.26, 1.97)	1.50 (1.19, 1.88)
	Low	5.85 (4.68, 7.31)	2.71 (2.16, 3.41)	2.14 (1.69, 2.69)	1.82 (1.44, 2.29)
Summed SES score	High [3]	1 (Ref)	1 (Ref)	1 (Ref)	1 (Ref)
	Medium [1-2]	3.64 (2.82, 4.70)	2.09 (1.62, 2.71)	1.80 (1.39, 2.34)	1.67 (1.28, 2.16)
	Low [0]	4.55 (3.28, 6.31)	4.81 (3.47, 6.68)	3.14 (2.25, 4.39)	2.40 (1.72, 3.36)
**T2D and CKD**					
LCA-defined SES	High	1 (Ref)	1 (Ref)	1 (Ref)	1 (Ref)
	Medium	2.64 (1.81, 3.85)	1.92 (1.31, 2.81)	1.63 (1.11, 2.38)	1.54 (1.05, 2.25)
	Low	6.49 (4.47, 9.42)	3.45 (2.35, 5.06)	2.27 (1.54, 3.34)	1.92 (1.30, 2.83)
Summed SES score	High [3]	1 (Ref)	1 (Ref)	1 (Ref)	1 (Ref)
	Medium [1-2]	3.68 (2.42, 5.59)	2.34 (1.53, 3.57)	1.78 (1.16, 2.72)	1.70 (1.11, 2.60)
	Low [0]	4.46 (2.60, 7.65)	4.31 (2.51, 7.39)	2.11 (1.22, 3.66)	1.84 (1.06, 3.20)
**CVD and T2D and CKD**					
LCA-defined SES	High	1 (Ref)	1 (Ref)	1 (Ref)	1 (Ref)
	Medium	3.66 (2.31, 5.80)	2.53 (1.59, 4.02)	2.21 (1.39, 3.52)	2.07 (1.30, 3.29)
	Low	10.67 (6.78, 16.81)	5.30 (3.34, 8.41)	3.49 (2.19, 5.56)	2.78 (1.74, 4.43)
Summed SES score	High [3]	1 (Ref)	1 (Ref)	1 (Ref)	1 (Ref)
	Medium [1-2]	5.34 (3.19, 8.93)	3.14 (1.87, 5.27)	2.41 (1.43, 4.06)	2.17 (1.29, 3.66)
	Low [0]	9.95 (5.55, 17.86)	10.38 (5.78, 18.63)	4.78 (2.64, 8.66)	3.22 (1.77, 5.84)

Model 1: unadjusted.

Model 2: adjusted for age at baseline, sex and ethnicity.

Model 3: model 2 also adjusted for BMI at baseline, current smoking and drinking status, healthy diet, sleep duration and physical activity.

Model 4: model 3 also adjusted for hypertension, dyslipidemia, medical history, COPD and cancer at baseline.

Abbreviations: SES, Socioeconomic status; LCA, Latent class analysis; HR, Hazard ratio; CI, Confidence interval; CVD, Cardiovascular diseases; T2D, Type 2 diabetes; CKD, Chronic kidney disease; CRMM, Cardio-renal-metabolic multimorbidity .

All P value are statistically significant ($P < 0.001$).

Table S5. Associations of income, education and employment status with cardio-renal-metabolic diseases (n=394,208).

	HR (95% CI)			
	**CVD**	**T2D**	**CKD**	**CRMM**
**Total household income level before tax**				
> £100,000	1 (Ref)	1 (Ref)	1 (Ref)	1 (Ref)
£52,000–£100,000	1.05 (0.98, 1.13)	**1.33 (1.18, 1.50)**	**1.45 (1.15, 1.84)**	1.22 (0.98, 1.50)
£31,000–£51,999	**1.12 (1.04, 1.20)**	**1.49 (1.33, 1.67)**	**1.67 (1.33, 2.10)**	**1.53 (1.24, 1.87)**
£18,000–£30,999	**1.15 (1.08, 1.24)**	**1.69 (1.51, 1.90)**	**1.79 (1.43, 2.25)**	**1.69 (1.38, 2.07)**
< £18,000	**1.23 (1.15, 1.32)**	**1.84 (1.64, 2.07)**	**2.02 (1.61, 2.55)**	**1.87 (1.52, 2.29)**
**Education qualification**				
College or University degree	1 (Ref)	1 (Ref)	1 (Ref)	1 (Ref)
A levels/AS levels or equivalent	0.98 (0.94, 1.02)	**1.10 (1.03, 1.17)**	1.01 (0.90, 1.13)	1.00 (0.90, 1.11)
O levels/GCSEs or equivalent	**1.06 (1.02, 1.10)**	**1.10 (1.05, 1.16)**	**1.17 (1.07, 1.28)**	**1.12 (1.03, 1.22)**
CSEs or equivalent	**1.10 (1.03, 1.17)**	**1.21 (1.12, 1.31)**	1.17 (1.00, 1.37)	**1.24 (1.07, 1.43)**
NVQ or HND or HNC or equivalent	**1.08 (1.03, 1.13)**	**1.20 (1.12, 1.28)**	**1.22 (1.09, 1.37)**	**1.19 (1.07, 1.32)**
Other professional qualifications	**1.08 (1.03, 1.14)**	**1.12 (1.04, 1.21)**	**1.15 (1.02, 1.31)**	1.06 (0.94, 1.20)
None of the above	**1.12 (1.08, 1.16)**	**1.16 (1.10, 1.22)**	**1.16 (1.06, 1.26)**	**1.22 (1.12, 1.33)**
**Employment status**				
Employed	1 (Ref)	1 (Ref)	1 (Ref)	1 (Ref)
Unemployed	**1.26 (1.21, 1.32)**	**1.32 (1.25, 1.39)**	**1.34 (1.20, 1.49)**	**1.37 (1.25, 1.50)**

Adjusted for age at baseline, sex, ethnicity, BMI at baseline, current smoking and drinking status, healthy diet, sleep duration, physical activity, hypertension, dyslipidemia, medical history, COPD and cancer at baseline.

Abbreviations: HR, hazard ratio; CI, confidence interval; CVD, Cardiovascular diseases; T2D, Type 2 diabetes; CKD, Chronic kidney disease; CRMM, Cardio-renal-metabolic multimorbidity.

P values shown in bold are statistically significant ($P < 0.05$).

Table S6. Associations of different patterns of socioeconomic status with cardio-renal-metabolic diseases by excluding individuals with missing information on covariates (n=323,536).

	HR (95% CI)			
	**CVD**	**T2D**	**CKD**	**CRMM**
**LCA-defined SES**				
High	1 (Ref)	1 (Ref)	1 (Ref)	1 (Ref)
Medium	1.15 (1.11, 1.20)	1.38 (1.30, 1.47)	1.50 (1.32, 1.69)	1.59 (1.42, 1.79)
Low	1.36 (1.30, 1.42)	1.79 (1.67, 1.91)	1.92 (1.69, 2.18)	2.11 (1.87, 2.38)
Per score point	1.17 (1.15, 1.20)	1.32 (1.28, 1.36)	1.34 (1.27, 1.41)	1.40 (1.33, 1.47)
**Summed SES score**				
High [3]	1 (Ref)	1 (Ref)	1 (Ref)	1 (Ref)
Medium [1-2]	1.20 (1.15, 1.26)	1.50 (1.40, 1.61)	1.52 (1.33, 1.74)	1.77 (1.55, 2.03)
Low [0]	1.60 (1.50, 1.72)	2.08 (1.90, 2.28)	2.15 (1.79, 2.58)	2.59 (2.19, 3.05)
Per score point	1.13 (1.11, 1.15)	1.25 (1.22, 1.29)	1.26 (1.20, 1.32)	1.31 (1.25, 1.37)

Adjusted for age at baseline, sex, ethnicity, BMI at baseline, current smoking and drinking status, healthy diet, sleep duration, physical activity, hypertension, dyslipidemia, medical history, COPD and cancer at baseline.

Abbreviations: SES, Socioeconomic status; HR, hazard ratio; CI, confidence interval; LCA, Latent class analysis; CVD, Cardiovascular diseases; T2D, Type 2 diabetes; CKD, Chronic kidney disease; CRMM, Cardio-renal-metabolic multimorbidity.

All P values are statistically significant (P < 0.001).

Table S7. Associations of different patterns of socioeconomic status with cardio-renal-metabolic diseases by imputing missing covariates with multiple imputation (n=394,208).

	HR (95% CI)			
	**CVD**	**T2D**	**CKD**	**CRMM**
**LCA-defined SES**				
High	1 (Ref)	1 (Ref)	1 (Ref)	1 (Ref)
Medium	1.16 (1.11, 1.20)	1.37 (1.29, 1.45)	1.47 (1.32, 1.65)	1.55 (1.40, 1.73)
Low	1.37 (1.32, 1.43)	1.81 (1.71, 1.93)	1.86 (1.65, 2.08)	2.09 (1.87, 2.33)
Per score point	1.17 (1.15, 1.20)	1.34 (1.30, 1.37)	1.31 (1.25, 1.38)	1.40 (1.34, 1.46)
**Summed SES score**				
High [3]	1 (Ref)	1 (Ref)	1 (Ref)	1 (Ref)
Medium [1-2]	1.21 (1.16, 1.26)	1.52 (1.42, 1.62)	1.48 (1.31, 1.68)	1.74 (1.54, 1.97)
Low [0]	1.59 (1.50, 1.69)	2.17 (1.99, 2.36)	2.09 (1.78, 2.46)	2.59 (2.23, 3.00)
Per score point	1.14 (1.12, 1.16)	1.28 (1.25, 1.31)	1.27 (1.22, 1.33)	1.33 (1.28, 1.38)

Adjusted for age at baseline, sex, ethnicity, BMI at baseline, current smoking and drinking status, healthy diet, sleep duration, physical activity, hypertension, dyslipidemia, medical history, COPD and cancer at baseline.

Abbreviations: SES, Socioeconomic status; HR, hazard ratio; CI, confidence interval; LCA, Latent class analysis; CVD, Cardiovascular diseases; T2D, Type 2 diabetes; CKD, Chronic kidney disease; CRMM, Cardio-renal-metabolic multimorbidity.

All P values are statistically significant (P < 0.001).

Table S8. Associations of different patterns of socioeconomic status with cardio-renal-metabolic diseases by excluding outcome events that occurred in the first 180 days of follow-up (n=392,737).

	HR (95% CI)			
	**CVD**	**T2D**	**CKD**	**CRMM**
**LCA-defined SES**				
High	1 (Ref)	1 (Ref)	1 (Ref)	1 (Ref)
Medium	1.15 (1.11, 1.20)	1.36 (1.28, 1.44)	1.47 (1.31, 1.65)	1.51 (1.36, 1.69)
Low	1.36 (1.30, 1.42)	1.80 (1.69, 1.92)	1.85 (1.65, 2.08)	2.05 (1.83, 2.29)
Per score point	1.17 (1.15, 1.19)	1.34 (1.30, 1.37)	1.32 (1.25, 1.38)	1.39 (1.33, 1.46)
**Summed SES score**				
High [3]	1 (Ref)	1 (Ref)	1 (Ref)	1 (Ref)
Medium [1-2]	1.49 (1.31, 1.68)	1.71 (1.51, 1.94)	1.21 (1.16, 1.26)	1.51 (1.41, 1.61)
Low [0]	2.08 (1.76, 2.45)	2.50 (2.14, 2.91)	1.57 (1.48, 1.67)	2.14 (1.96, 2.33)
Per score point	1.27 (1.22, 1.33)	1.32 (1.27, 1.38)	1.14 (1.12, 1.15)	1.27 (1.24, 1.30)

Adjusted for age at baseline, sex, ethnicity, BMI at baseline, current smoking and drinking status, healthy diet, sleep duration, physical activity, hypertension, dyslipidemia, medical history, COPD and cancer at baseline.

Abbreviations: SES, Socioeconomic status; HR, hazard ratio; CI, confidence interval; LCA, Latent class analysis; CVD, Cardiovascular diseases; T2D, Type 2 diabetes; CKD, Chronic kidney disease; CRMM, Cardio-renal-metabolic multimorbidity.

All P values are statistically significant ($P < 0.001$).

Table S9. Associations of different patterns of socioeconomic status with cardio-renal-metabolic diseases using the competing risk model (n = 394,208).

	HR (95% CI)			
	**CVD**	**T2D**	**CKD**	**CRMM**
**LCA-defined SES**				
Low	1 (Ref)	1 (Ref)	1 (Ref)	1 (Ref)
Medium	1.14 (1.10, 1.18)	1.36 (1.28, 1.44)	1.44 (1.29, 1.61)	1.53 (1.37, 1.70)
High	1.32 (1.27, 1.37)	1.74 (1.63, 1.85)	1.75 (1.56, 1.97)	1.97 (1.77, 2.21)
Per score point	1.15 (1.13, 1.17)	1.31 (1.27, 1.34)	1.27 (1.21, 1.34)	1.35 (1.29, 1.42)
**Summed SES score**				
High [3]	1 (Ref)	1 (Ref)	1 (Ref)	1 (Ref)
Medium [1-2]	1.19 (1.14, 1.24)	1.49 (1.39, 1.59)	1.44 (1.28, 1.63)	1.70 (1.51, 1.93)
Low [0]	1.45 (1.36, 1.55)	1.98 (1.81, 2.16)	1.82 (1.54, 2.15)	2.28 (1.95, 2.65)
Per score point	1.12 (1.10, 1.14)	1.25 (1.22, 1.28)	1.23 (1.18, 1.29)	1.28 (1.23, 1.34)

Adjusted for age at baseline, sex, ethnicity, BMI at baseline, current smoking and drinking status, healthy diet, sleep duration, physical activity, hypertension, dyslipidemia, medical history, COPD and cancer at baseline.

Abbreviations: SES: Socioeconomic status; LCA: Latent class analysis; CRMM: Cardio-renal-metabolic multimorbidity; CVD: Cardiovascular diseases; T2D: Type 2 diabetes; CKD: Chronic kidney disease.

All P values are statistically significant ($P < 0.001$).

Table S10. Associations of Townsend deprivation index with cardio-renal-metabolic diseases (n=394,208).

**TDI**	***HR (95% CI)***			
	**CVD**	**T2D**	**CKD**	**CRMM**
T_1	1 (Ref)	1 (Ref)	1 (Ref)	1 (Ref)
T_2	1.03 (1.00, 1.06)	1.14 (1.10, 1.20)	1.09 (1.01, 1.17)	1.16 (1.08, 1.24)
T_3	1.08 (1.05, 1.11)	1.33 (1.28, 1.39)	1.14 (1.06, 1.23)	1.32 (1.23, 1.41)
Per score point	1.04 (1.03, 1.06)	1.16 (1.13, 1.18)	1.07 (1.03, 1.11)	1.15 (1.11, 1.19)

Adjusted for age at baseline, sex, ethnicity, BMI at baseline, current smoking and drinking status, healthy diet, sleep duration, physical activity, hypertension, dyslipidemia, medical history, COPD and cancer at baseline.

T_1 : low socioeconomic deprivation; T_2 : medium socioeconomic deprivation; T_3 : high socioeconomic deprivation.

Abbreviations: TDI, Townsend deprivation index; HR, hazard ratio; CI, confidence interval; CVD, Cardiovascular diseases; T2D, Type 2 diabetes; CKD, Chronic kidney disease; CRMM, Cardio-renal-metabolic multimorbidity.

All P values are statistically significant ($P < 0.05$).

Table S11. Correlation between different patterns of socioeconomic status and Townsend deprivation index (n = 394,208).

	Low TDI (n=132,907)	Medium TDI (n=131,880)	High TDI (n=129,421)	Cramer's V	*P*
**LCA-defined SES**				0.140	<0.001
High	33,190 (25.0)	26,310 (19.9)	20,356 (15.7)		
Medium	76,221 (57.3)	73,391 (55.7)	59,421 (45.9)		
Low	23,496 (17.7)	32,179 (24.4)	49,644 (38.4)		
**Summed SES score**				0.090	<0.001
High [3]	23,747 (17.9)	19,340 (14.7)	15,934 (12.3)		
Medium [1-2]	105,797 (79.6)	107,999 (81.9)	103,006 (79.6)		
Low [0]	3,363 (2.5)	4,541 (3.4)	10,481 (8.1)		

Abbreviations: SES, Socioeconomic status; TDI, Townsend deprivation index; LCA: Latent class analysis.

Table S12. Associations of different patterns of socioeconomic status with cardio-renal-metabolic diseases after adjusting Townsend deprivation index (n = 394,208).

	HR (95% CI)			
	**CVD**	**T2D**	**CKD**	**CRMM**
**LCA-defined SES**				
Low	1 (Ref)	1 (Ref)	1 (Ref)	1 (Ref)
Medium	1.15 (1.11, 1.20)	1.35 (1.28, 1.44)	1.47 (1.31, 1.64)	1.54 (1.38, 1.72)
High	1.35 (1.30, 1.41)	1.72 (1.61, 1.83)	1.81 (1.61, 2.03)	1.99 (1.78, 2.23)
Per score point	1.17 (1.14, 1.19)	1.30 (1.26, 1.33)	1.30 (1.23, 1.36)	1.36 (1.30, 1.42)
**Summed SES score**				
High [3]	1 (Ref)	1 (Ref)	1 (Ref)	1 (Ref)
Medium [1-2]	1.20 (1.15, 1.25)	1.20 (1.15, 1.25)	1.46 (1.30, 1.66)	1.71 (1.51, 1.93)
Low [0]	1.55 (1.46, 1.65)	1.55 (1.46, 1.65)	2.01 (1.7, 2.36)	2.41 (2.07, 2.80)
Per score point	1.13 (1.11, 1.15)	1.25 (1.22, 1.28)	1.26 (1.20, 1.31)	1.30 (1.25, 1.35)

Adjusted for age at baseline, sex, ethnicity, BMI at baseline, current smoking and drinking status, healthy diet, sleep duration, physical activity, hypertension, dyslipidemia, medical history, COPD and cancer at baseline.

Abbreviations: SES, Socioeconomic status; TDI, Townsend deprivation index; HR, hazard ratio; CI, confidence interval; LCA: Latent class analysis; CVD: Cardiovascular diseases; T2D: Type 2 diabetes; CKD: Chronic kidney disease; CRMM: Cardio-renal-metabolic multimorbidity.

All P values are statistically significant (P < 0.001).

Table S13. Associations of patterns of socioeconomic status defined by latent class analysis with cardio-renal-metabolic diseases, stratified by Townsend deprivation index (n = 394,208).

TDI	LCA-defined SES	*HR (95% CI)* CVD	T2D	CKD	CRMM
T_1	Medium	1.36 (1.28, 1.44)	1.28 (1.17, 1.41)	1.83 (1.54, 2.18)	1.78 (1.50, 2.12)
	Low	1.80 (1.69, 1.93)	1.64 (1.47, 1.82)	2.72 (2.26, 3.28)	2.58 (2.14, 3.11)
T_2	Medium	1.43 (1.34, 1.53)	1.46 (1.32, 1.62)	1.98 (1.63, 2.41)	1.90 (1.58, 2.29)
	Low	1.90 (1.77, 2.04)	1.81 (1.63, 2.01)	3.04 (2.48, 3.72)	2.81 (2.32, 3.40)
T_3	Medium	1.31 (1.22, 1.42)	1.48 (1.33, 1.65)	1.71 (1.37, 2.13)	1.69 (1.38, 2.07)
	Low	1.93 (1.79, 2.08)	2.00 (1.79, 2.23)	2.53 (2.03, 3.14)	2.62 (2.14, 3.20)

Adjusted for age at baseline, sex, ethnicity, BMI at baseline, current smoking and drinking status, healthy diet, sleep duration, physical activity, hypertension, dyslipidemia, medical history, COPD and cancer at baseline.

T_1 : low socioeconomic deprivation; T_2 : medium socioeconomic deprivation; T_3 : high socioeconomic deprivation.

Abbreviations: LCA, Latent class analysis; SES, Socioeconomic status; CVD, Cardiovascular diseases; T2D, Type 2 diabetes; CKD, Chronic kidney disease; CRMM, Cardio-renal-metabolic multimorbidity; TDI, Townsend deprivation index; HR, hazard ratio; CI, confidence interval.

All P values are statistically significant ($P < 0.001$).

Table S14. Associations of patterns of summed socioeconomic status score with cardio-renal-metabolic diseases, stratified by Townsend deprivation index (n = 394,208).

TDI	Summed SES score	*HR (95% CI)* CVD	T2D	CKD	CRMM
T_1	Medium [1-2]	1.39 (1.31, 1.48)	1.41 (1.27, 1.57)	1.83 (1.51, 2.21)	1.98 (1.63, 2.41)
	Low [0]	1.43 (1.25, 1.64)	1.64 (1.35, 1.99)	1.84 (1.31, 2.57)	2.14 (1.55, 2.97)
T_2	Medium [1-2]	1.53 (1.43, 1.65)	1.61 (1.44, 1.81)	2.18 (1.75, 2.71)	2.19 (1.77, 2.71)
	Low [0]	1.68 (1.49, 1.89)	2.13 (1.80, 2.51)	2.23 (1.63, 3.04)	2.33 (1.74, 3.12)
T_3	Medium [1-2]	1.55 (1.43, 1.68)	1.66 (1.47, 1.88)	1.90 (1.51, 2.40)	2.17 (1.73, 2.72)
	Low [0]	1.66 (1.50, 1.83)	2.28 (1.99, 2.61)	1.87 (1.42, 2.44)	2.50 (1.95, 3.20)

Adjusted for age at baseline, sex, ethnicity, BMI at baseline, current smoking and drinking status, healthy diet, sleep duration, physical activity, hypertension, dyslipidemia, medical history, COPD and cancer at baseline.

T_1 : low socioeconomic deprivation; T_2 : medium socioeconomic deprivation; T_3 : high socioeconomic deprivation.

Abbreviations: SES, Socioeconomic status; CVD, Cardiovascular diseases; T2D, Type 2 diabetes; CKD, Chronic kidney disease; CRMM, Cardio-renal-metabolic multimorbidity; TDI, Townsend deprivation index; HR, hazard ratio; CI, confidence interval; .

All P values are statistically significant ($P < 0.001$).

Table S15. Association of socioeconomic status defined by latent class analysis with the disease progression, using different intervals for the participants entering different states on the same date (n=394,208).

	HR (95% CI)		
	**30 days**	**180 days**	**365 days**
**Free of CRMD to FCRMD**			
High	1 (Ref)	1 (Ref)	1 (Ref)
Medium	**1.2086 (1.1709, 1.2476)**	**1.2077 (1.1699, 1.2466)**	**1.2069 (1.1692, 1.2458)**
Low	**1.4453 (1.3969, 1.4953)**	**1.4449 (1.3966, 1.4950)**	**1.4431 (1.3948, 1.4931)**
**Free of CRMD to Death**			
High	1 (Ref)	1 (Ref)	1 (Ref)
Medium	**1.1349 (1.0796, 1.1930)**	**1.1352 (1.0799, 1.1933)**	**1.1353 (1.0800, 1.1934)**
Low	**1.5063 (1.4283, 1.5886)**	**1.5069 (1.4289, 1.5892)**	**1.5087 (1.4307, 1.5911)**
**FCRMD to CRMM**			
High	1 (Ref)	1 (Ref)	1 (Ref)
Medium	**1.3188 (1.1844, 1.4685)**	**1.3121 (1.1776, 1.4620)**	**1.3061 (1.1714, 1.4562)**
Low	**1.4985 (1.3427, 1.6724)**	**1.4986 (1.3419, 1.6737)**	**1.4891 (1.3325, 1.6642)**
**FCRMD to Death**			
High	1 (Ref)	1 (Ref)	1 (Ref)
Medium	**1.1428 (1.0297, 1.2684)**	**1.1421 (1.0290, 1.2676)**	**1.1407 (1.0278, 1.2661)**
Low	**1.3468 (1.2107, 1.4982)**	**1.3446 (1.2087, 1.4958)**	**1.3419 (1.2063, 1.4928)**
**CRMM to Death**			
High	1 (Ref)	1 (Ref)	1 (Ref)
Medium	1.2018 (0.9382, 1.5395)	1.2234 (0.9533, 1.5700)	1.2382 (0.9630, 1.5920)
Low	**1.4049 (1.0956, 1.8015)**	**1.4220 (1.1071, 1.8265)**	**1.4290 (1.1103, 1.8392)**

Adjusted for age at baseline, sex, ethnicity, BMI at baseline, current smoking and drinking status, healthy diet, sleep duration, physical activity, COPD and cancer at baseline.

Abbreviations: LCA, Latent class analysis; SES, Socioeconomic status; HR, Hazard ratio; CI, Confidence interval; CRMD, Cardio-renal-metabolic disease; FCRMD, First cardio-renal-metabolic disease; CRMM, Cardio-renal-metabolic multimorbidity (the coexistence of two or three CRMDs after FCRMD).

P values shown in bold are statistically significant ($P < 0.05$).

Table S16. Association of summed socioeconomic status score with the disease progression, using different intervals for the participants entering different states on the same date (n=394,208).

	HR (95% CI)		
	**30 days**	**180 days**	**365 days**
**Free of CRMD to FCRMD**			
High [3]	1 (Ref)	1 (Ref)	1 (Ref)
Medium [1-2]	**1.2468 (1.2039, 1.2913)**	**1.2466 (1.2036, 1.2911)**	**1.2448 (1.2019, 1.2893)**
Low [0]	**1.7919 (1.7050, 1.8832)**	**1.4872 (1.0955, 2.0191)**	**1.7874 (1.7006, 1.8786)**
**Free of CRMD to Death**			
High [3]	1 (Ref)	1 (Ref)	1 (Ref)
Medium [1-2]	**1.2101 (1.1454, 1.2786)**	**1.2104 (1.1456, 1.2788)**	**1.2103 (1.1455, 1.2786)**
Low [0]	**2.0174 (1.8665, 2.1806)**	**1.7910 (1.7041, 1.8823)**	**2.0249 (1.8736, 2.1884)**
**FCRMD to CRMM**			
High [3]	1 (Ref)	1 (Ref)	1 (Ref)
Medium [1-2]	**1.4088 (1.2466, 1.5921)**	**1.4104 (1.2466, 1.5956)**	**1.3986 (1.2356, 1.5831)**
Low [0]	**1.5884 (1.3678, 1.8446)**	**1.5932 (1.3703, 1.8523)**	**1.5800 (1.3579, 1.8384)**
**FCRMD to Death**			
High	1 (Ref)	1 (Ref)	1 (Ref)
Medium	**1.2414 (1.1033, 1.3969)**	**1.2399 (1.1019, 1.3952)**	**1.2384 (1.1005, 1.3935)**
Low	**1.7613 (1.5194, 2.0416)**	**1.7598 (1.5181, 2.0399)**	**1.7555 (1.5143, 2.0351)**
**CRMM to Death**			
High	1 (Ref)	1 (Ref)	1 (Ref)
Medium	**1.4614 (1.0801, 1.9774)**	**1.4872 (1.0955, 2.0191)**	**1.5375 (1.1285, 2.0946)**
Low	**1.9235 (1.3689, 2.7027)**	**1.9369 (1.3737, 2.7309)**	**1.9571 (1.3821, 2.7715)**

Adjusted for age at baseline, sex, ethnicity, BMI at baseline, current smoking and drinking status, healthy diet, sleep duration, physical activity, hypertension, dyslipidemia, medical history, COPD and cancer at baseline.

Abbreviations: SES, Socioeconomic status; HR, hazard ratio; CI, confidence interval; CRMD, Cardio-renal-metabolic disease; FCRMD, First cardio-renal-metabolic disease; CRMM, Cardio-renal-metabolic multimorbidity (the coexistence of two or three CRMDs after FCRMD).

P values shown in bold are statistically significant ($P < 0.05$).

Supplementary Figures

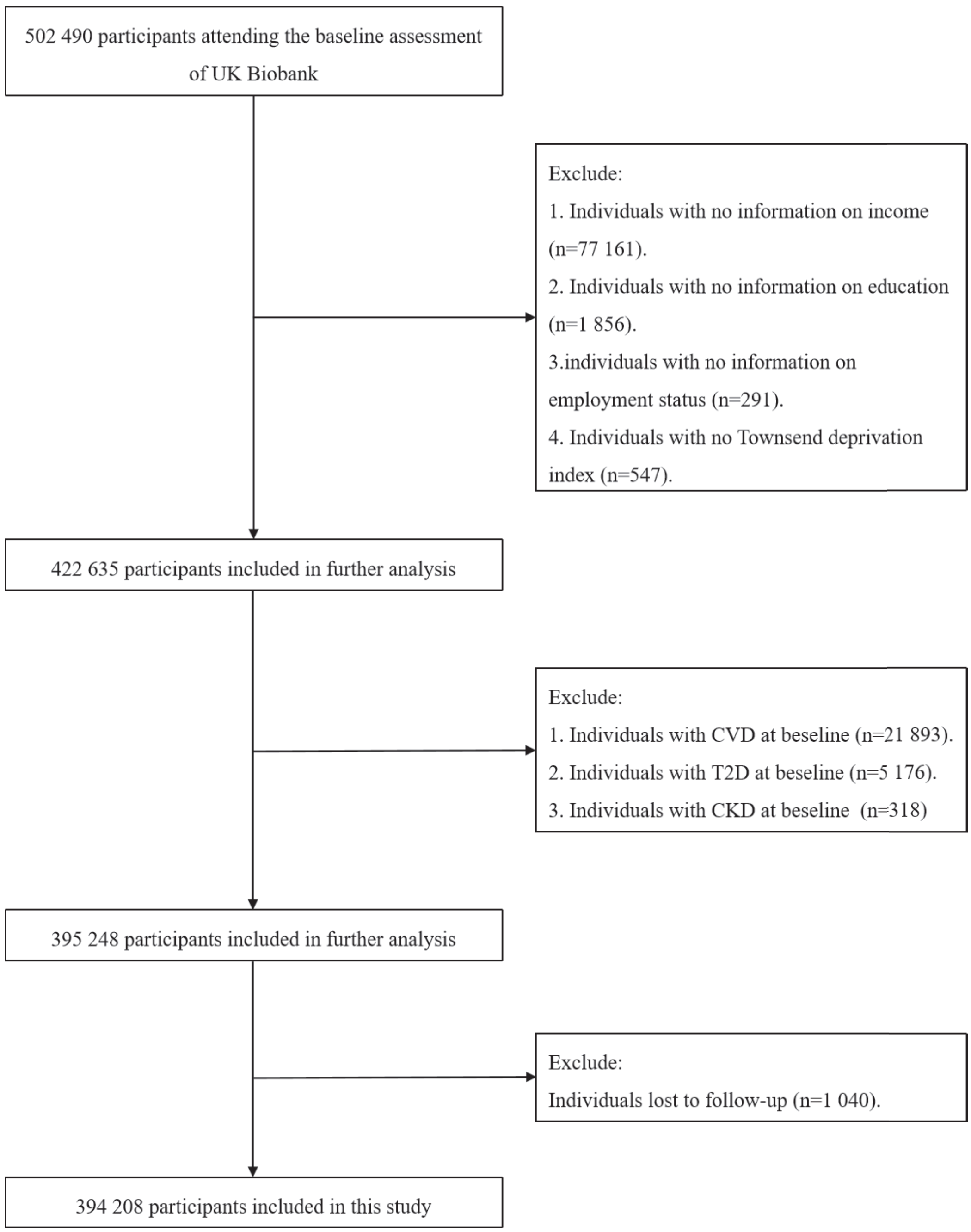

Figure S1. Flow chart of the selection process.

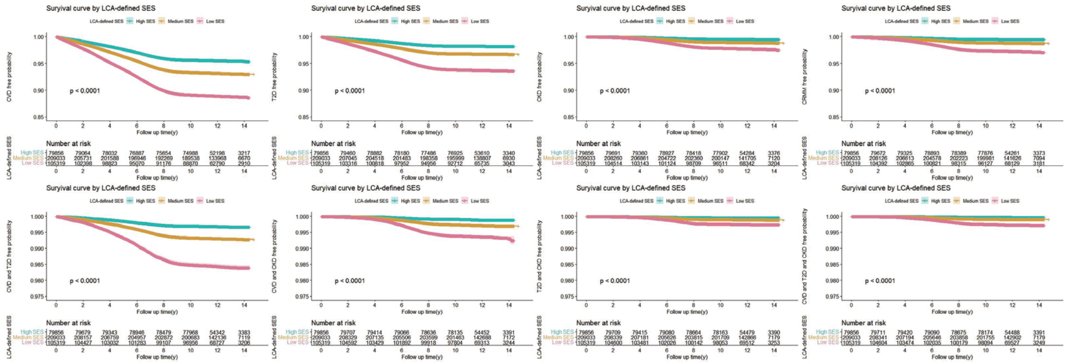

Figure S2. The Kaplan-Meier curve for different outcomes across socioeconomic status defined by latent class analysis.

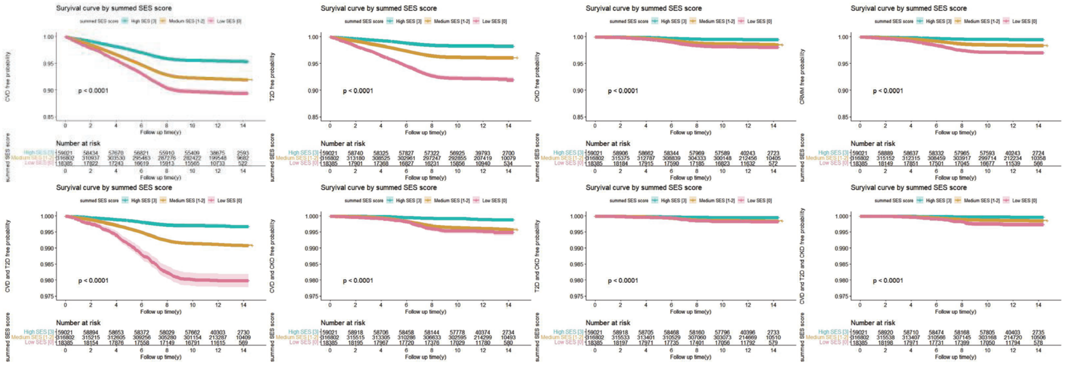

Figure S3. The Kaplan-Meier curve for different outcomes across summed socioeconomic status score.

References

1. Zhang Y-B, Chen C, Pan X-F, et al. Associations of healthy lifestyle and socioeconomic status with mortality and incident cardiovascular disease: Two prospective cohort studies. *BMJ*. 2021:n604.

2. Ye X, Wang Y, Zou Y, et al. Associations of socioeconomic status with infectious diseases mediated by lifestyle, environmental pollution and chronic comorbidities: A comprehensive evaluation based on UK biobank. *Infect Dis Poverty*. 2023;12:5.

3. Yang S, Xiao Y, Jing D, et al. Socioeconomic disparity in the natural history of cutaneous melanoma: Evidence from two large prospective cohorts. *J Epidemiol Community Health*. 2024;78:713–720.

4. Van Horn L, Carson JAS, Appel LJ, et al. Recommended dietary pattern to achieve adherence to the American heart association/american college of cardiology (AHA/ACC) guidelines: A scientific statement from the american heart association. *Circulation*. 2016;134.

5. Chaput J-P, Dutil C, Sampasa-Kanyinga H. Sleeping hours: What is the ideal number and how does age impact this? *NSS*. 2018;10:421–430.
